# Optogenetically-Induced Population Discharge Threshold as a Sensitive Measure of Network Excitability

**DOI:** 10.1523/ENEURO.0229-18.2019

**Published:** 2019-11-06

**Authors:** D. C. Klorig, G. E. Alberto, T. Smith, D. W. Godwin

**Affiliations:** 1 Department of Neurobiology and Anatomy; 2 Neuroscience Program; 3 Department of Physiology and Pharmacology, Wake Forest University, Winston-Salem, NC

**Keywords:** epilepsy, excitability, intensity response, network, optogenetics, seizure

## Abstract

Network excitability is governed by synaptic efficacy, intrinsic excitability, and the circuitry in which these factors are expressed. The complex interplay between these factors determines how circuits function and, at the extreme, their susceptibility to seizure. We have developed a sensitive, quantitative estimate of network excitability in freely behaving mice using a novel optogenetic intensity-response procedure. Synchronous activation of deep sublayer CA1 pyramidal cells produces abnormal network-wide epileptiform population discharges (PDs) that are nearly indistinguishable from spontaneously-occurring interictal spikes (IISs). By systematically varying light intensity, and therefore the magnitude of the optogenetically-mediated current, we generated intensity-response curves using the probability of PD as the dependent variable. Manipulations known to increase excitability, such as sub-convulsive doses (20 mg/kg) of the chemoconvulsant pentylenetetrazol (PTZ), produced a leftward shift in the curve compared to baseline. The anti-epileptic drug levetiracetam (LEV; 40 mk/kg), in combination with PTZ, produced a rightward shift. Optogenetically-induced PD threshold (oPDT) baselines were stable over time, suggesting the metric is appropriate for within-subject experimental designs with multiple pharmacological manipulations.

## Significance Statement

Abnormal excitability is associated with a number of neurologic disorders, including epilepsy. Excitability can be measured in single cells *in vitro*, but it is difficult to extrapolate from these values to the functional impact on the associated network. Epileptiform population discharges (PDs) are network-wide events that represent a distinct transition from normal to abnormal functional modes. We developed a new method that uses light intensity-response curves to precisely determine the threshold for this transition as a surrogate measure of network excitability.

## Introduction

Seizure thresholds are commonly used to determine excitability and seizure susceptibility in animal models of epilepsy and to assess the effectiveness of therapeutic intervention (Spiegel, 1937; Ziskind et al., 1946; Swinyard et al., 1952; Barton et al., 2001). However, seizures have lasting effects on the brain, including widespread changes in gene expression (Altar et al., 2004), reduced seizure thresholds, and increased seizure severity (Racine, 1972a). This complicates the interpretation of within-subject experiments and limits the ability to make multiple measurements over time. Furthermore, excitability is dynamically modulated by ongoing brain activity and behavioral state, which introduces significant variability, limiting the precision of acute threshold measurements. Single pulse electrical stimulation, a technique used in humans undergoing intraoperative monitoring, provides a measure of network excitability without inducing seizure (Matsumoto et al., 2017). However, this method is limited by the uncertainty of the cells and pathways stimulated (Histed et al., 2009), and the ambiguity of amplitude based LFP measurements in relation to underlying activity (Hales and Pockett, 2014; Herreras, 2016). Excitability and seizure susceptibility can also be estimated by quantification of spontaneous seizures in models of epileptogenesis, but this approach is complicated by the unpredictable occurrence of seizures, requiring constant video-EEG monitoring, prolonged drug administration, and large numbers of animals (Löscher, 2011).

To obtain more precise and reliable measurements, we have developed a rigorous approach for quantifying instantaneous network excitability, without inducing seizures, using single pulse light intensity-response curves to determine population discharge (PD) thresholds. Optogenetically-induced PDs (oPDs) are all-or-none, network-wide events with waveforms and latencies similar to spontaneously-occurring interictal spikes (IISs). We show that induced oPDs occur with a higher likelihood with increasing light intensity. By delivering a range of light intensities with randomized presentation order and modeling oPD probability using logistic regression, we account for short-term fluctuations in excitability and derive a new metric, the I50, defined as the intensity of light that produces an oPD with a probability of 0.5. Using established pharmacologic modulators, we demonstrate that the I50 is sensitive to shifts in excitability. The oPD threshold (oPDT) can be monitored over long timescales with high temporal resolution to fully capture the dynamics of network excitability. Furthermore, because it does not involve the generation of after-discharges (ADs) or seizures, the oPDT can be used as a measure of excitability in a range of normal and pathologic conditions other than epilepsy.

Using the same preparation, we show that repetitive stimulation with light of a sufficient intensity produces a robust AD and kindling with daily ADs results in overt behavioral seizure. In the same animal, the optogenetic AD threshold (oADT) and the oPDT are equivalent in terms of light intensity. The oADT, however, requires an additional accumulating process involving multiple oPDs in a short period of time. Comparing the two metrics allows for separation of the contribution of instantaneous excitability, and robustness to seizure, or susceptibility, features that are inseparably intertwined in other measures of seizure threshold.

## Materials and Methods

### Subjects

Male Thy1-ChR2-YFP (founder line 18) transgenic mice (Arenkiel et al., 2007; Wang et al., 2007; stock #007612, The Jackson Laboratory) were used for chronic optogenetic stimulation and recording (*n* = 35). Thy1 line 18 mice express wild-type ChR2 fused to EYFP in area CA1, subiculum (Sub), and layer 5 of cortex (Arenkiel et al., 2007). In area CA1, expression is specific to excitatory pyramidal neurons and is concentrated in calbindin-negative cells in the deep pyramidal cell sublayer (Dobbins et al., 2018). These mice can be bred as homozygotes, ensuring consistent expression levels and patterns from animal to animal, a significant advantage for reproducibility. For this reason, they are used in a number of optogenetics studies (Ting and Feng, 2013). Not all animals were used for all experiments; exact numbers are reported throughout. All animal experiments were approved by the Institutional Animal Care and Use Committee (IACUC) of Wake Forest University in agreement with National Institutes of Health and United States Department of Agriculture guidelines.

### Chronic implant

**Figure 1.**
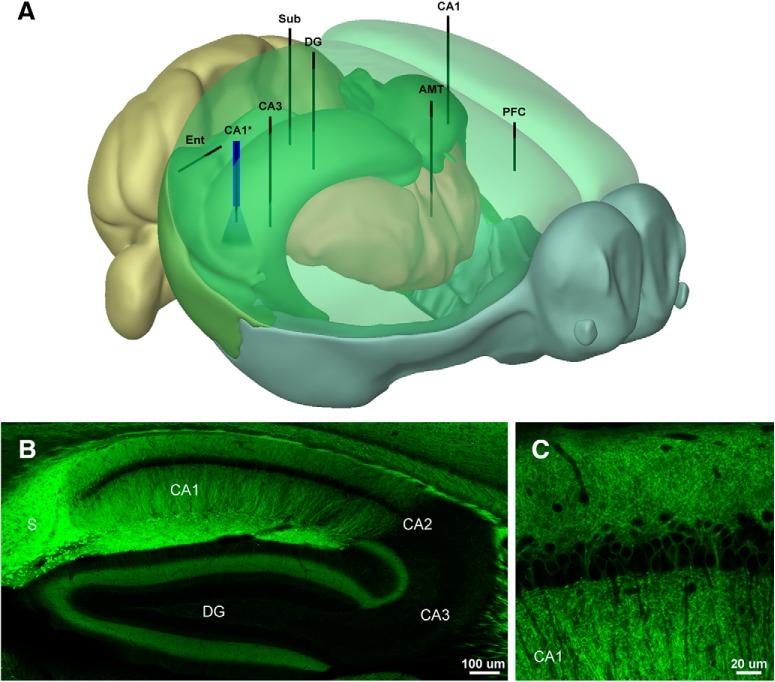
Electrode placement and expression patterns in the Thy1-ChR2 (line 18) mouse. ***A***, Schematic showing the placement of the electrode array and optrode. ***B***, Distribution of ChR2-EYFP in the hippocampus of the Thy1 (line 18) mouse. Note high expression levels in CA1 and Sub; 10× tiled confocal image. ***C***, ChR2-EYFP is expressed in deep pyramidal neurons in CA1 (Dobbins et al., 2018); 63× oil immersion confocal image. The 3D model was generated using Brain Explorer Software courtesy of the Allen Brain Institute (http://mouse.brain-map.org/static/brainexplorer).

Details of the implantation surgery and chronic recording array are described in a previous publication (Klorig and Godwin, 2014). Briefly, Thy1 mice (age two to nine months, mean 120 ± 9 d) were anesthetized with isoflurane and placed in a stereotaxic device. All surgical procedures were performed under red light to avoid sustained activation of ChR2. Metal ground screws were secured to the cranium bilaterally so that they were in contact with the subarachnoid space posterior to the transverse sinus. A total of 8 tungsten microwires were implanted in a satellite array in cortical, subcortical, and hippocampal locations: prefrontal cortex (PFC; AP: 5.58 mm, L: 0.37 mm, DV: –1.81 mm, from the interaural plane), anterior medial thalamic nucleus (AMTh; AP: 2.86 mm, L: 0.24 mm, DV: –3.62 mm), dentate gyrus (DG; AP: 1.50 mm, L: 1.39 mm, DV: –1.61 mm), hippocampus (CA3; AP: 1.34 mm, L: 2.88 mm, DV: –2.12 mm), entorhinal cortex (Ent; AP: 0.19 mm, L: 3.90 mm, DV: –2.73 mm, angled 16° posterior), Sub (AP: 0.88 mm, L: 1.02 mm, DV: –1.43 mm), and left CA1 (AP: 1.00 mm, L: –2.36 mm, DV: –1.20 mm; Fig. 1*A*). A magnetic rotary fiber connector with an attached microwire (optrode) was placed in intermediate CA1 near Sub (CA1a; AP: 1.00 mm, L: 2.36 mm, DV: –1.20 mm, from the interaural plane). The stimulation fiber was a multimodal fiber with a 200 µm diameter core and a numerical aperture of 0.39 (FT200UMT, ThorLabs). The recording electrode extended 0.5 mm beyond the fiber tip. The optical fiber was situated 0.4 mm dorsal to the pyramidal cell layer and 0.3 mm dorsal to the basal dendrites. The optrode was configured to minimize the photovoltaic effect (<0.02% LFP amplitude) by cutting the electrode at an angle so that the exposed metal surface was not directly illuminated by the implanted fiber (Cardin et al., 2010). Wild-type (*n* = 2) and postmortem control (*n* = 4) recordings were used to confirm the absence of artifact. This setup allowed for high quality recordings, free of movement artifacts, even during behavioral seizures.

### Light stimulation and recordings

Mice were allowed to recover one week before recordings. Wideband (0.3–20 kHz, sampled at 40 kHz) depth recordings were made using the SciWorks recording system (DataWave Technologies), AM-3600 extracellular amplifiers (A-M Systems), and T8G100 headstage amplifiers (TBSI). Photostimulation was performed with a fiber-coupled LED with peak emission at 470 nm (M470F3, ThorLabs). LED intensity was controlled using an LED driver with analog modulation (LEDD1B, ThorLabs). Analog output pulses were generated within SciWorks and used to control the LED driver. Light pulses were continually monitored with a silicon photodiode placed near the emitter. A custom op-amp based current-to-voltage converter circuit was used to linearize the photodiode output and the resulting signals were recorded along with the electrophysiology data. Two measures of light output are provided; the power (mW) at the tip of the source fiber and an arbitrary linearized scale. Light power ranged from 0.43 to 4.15 mW at the fiber tip corresponding to an irradiance of 0.2–1.96 mW/mm^2^ at the recording site estimated using the empirically derived model described in (Aravanis et al., 2007; Yizhar et al., 2011).

### Video and behavioral scoring

Recording sessions were video recorded and an infrared LED synchronized to the stimulus onset was placed in view of the video camera for precise temporal alignment between the video and the recordings. Video was used for assessment of behavioral seizures using a modified Racine scale (Racine, 1972b; Pinel and Rovner, 1978). Latencies to seizure stage relative to the onset of the stimulus and durations were also recorded.

### Histologic verification of electrode placement

On completion of recording experiments, mice were anesthetized with Euthasol (Virbac Animal Health), electrolytic lesions were performed at each electrode site (20 nA, 10 s), then animals were transcardially perfused with saline and 4% paraformaldehyde solution in PB. After removal and postfixing, brains were sectioned in 50-µm slices using a vibratome. The slices were then mounted on slides and stained using cresyl violet (Nissl). Each slice was imaged using a light microscope and the stereotaxic coordinates of the implanted electrodes were recorded. Animals were excluded from further analysis if electrodes used in those analyses were located outside of the target areas (*n* = 5).

### Confocal microscopy

ChR2-eYFP expression in Thy1 mice was imaged in non-implanted animals. Confocal imaging was performed on a Zeiss LSM 710 confocal microscope with 10×, 20× air, and 60× water immersion objectives.

### Experimental design and statistical analysis

Data analysis was performed using custom scripts written in MATLAB (MathWorks). The suite of stimulus presentation and analysis tools used to generate and calculate the oPDT is available on github (https://github.com/neuroptics/optoDR).

### oPD intensity-response curve

Single square pulses (10 ms) of light were delivered at a range of intensities (20 levels, 0.43–4.15 mW at fiber tip, 0.2–1.96 mW/mm^2^ at the recording site). Sixty repetitions of each intensity were presented in randomly ordered blocks with a 1- or 3-s interval between pulses. A timing pulse was used to precisely align responses to the onset of the LED. For the PD curves, time-locked evoked responses were extracted, the 2nd derivative calculated, and a sliding window (10 ms) was used to calculate root-mean-square (RMS) power via convolution. The max RMS within a specified time window was calculated, sorted by magnitude, and plotted for each channel over all stimulation levels. The channel with the sharpest transition between sub-PD and PD was chosen and the threshold set (typically contralateral CA1, but occasionally DG or CA3 were chosen). PD detection was verified visually. All subsequent analyses for a given animal were performed with the same channel and threshold. The ratio of PD events per level was calculated to generate a probability curve. This curve was fit by the Hodgkin and Huxley formulation of the Boltzmann distribution:

$$f\left(x\right)=\frac{1}{1+e^{\left(\frac{a-x}{b}\right)}},$$

where a is the I50, and b is the slope (Hodgkin and Huxley, 1952), using nonlinear regression (least squares) in MATLAB. The I50 value for each session was then used for further comparisons.

### oADT and kindling procedure

oADTs were determined by presenting optical stimulation trains at 6.67 Hz (10-s duration, 4-ms pulse, 150-ms interval, 66 pulses), then increasing the intensity (with a 2-min interval) until an AD was evoked (characterized by high amplitude, self-sustaining activity that persisted > 5 s after the stimulus ended). A modified optogenetic kindling procedure was used that allowed for estimation of oADTs, involving repeated daily light stimulation at increasing intensity until an AD was evoked (with intensities identical to that performed on the first day), similar to established procedures (Pinel et al., 1976; Bragin et al., 2002). ADs were defined as sustained high-amplitude spike and poly-spike activity lasting at least 5 s following termination of the stimulus. oAD durations were measured from the start of the stimulus to the last spike of the oAD. Only one oAD was evoked per 24-h period. This procedure was repeated each day for 15 d.

### Pharmacology

All experiments used a within-subjects design. Treatments were compared to a pre-treatment baseline using repeated measures one-way ANOVA with Tukey’s test for multiple comparisons. Saline controls were also performed and compared to pre-injection baselines.

### Statistics and measures of reliability

Statistical testing was performed with MATLAB and Prism (GraphPad). All means are reported ± SD, with 95% confidence intervals (CI) where appropriate. Datasets were tested for normality using the omnibus K^2^ test (D’Agostino et al., 1990). Comparisons were performed using repeated measures one-way ANOVA with Tukey’s test for multiple comparisons, paired *t* tests, Fischer’s exact test, or Wilcoxon matched pairs test where appropriate. Correlation coefficients were calculated using Pearson’s or Spearman’s method where appropriate. All reported statistics are labeled with the test used. Box and whisker plots have min-max whiskers, 25th to 75th percentile boxes, and the central line is the median. The probability of oAD given at least × oPDs was calculated using the formula: $P(AD|\sum^{\;}PD_{i}\geq x)=\frac{P(AD\cap \sum^{\;}PD_{i}\geq x)}{P(\sum^{\;}PD_{i}\geq x)}$. For assessment of oAD probability given oPD on a given stimulus pulse *n*, we used the formula for conditional probability: $P(AD|PD_{n})=\frac{P(AD\cap PD_{n})}{P(PD_{n})}$. For assessment of oPD_I50_ probabilities given the results of previous trials we used the formula: $P(PD_{\text{I}50}|PD_{n-\text{back}})=\frac{P(PD_{I50}\cap PD_{n-\text{back}})}{P(PD_{n-\text{back}})}$. Fisher’s exact test was used to compare proportions.

## Results

To explore network propagation of optogenetically-induced seizures and PD activity, we developed a multi-site satellite array system consisting of individually placed microwires in perihippocampal structures and an optrode above CA1. Targets included PFC, AMTh, Ent, DG, Sub, hippocampal area CA3, and area CA1 bilaterally (CA1-L and CA1-R; Fig. 1*A*). These recording sites provide coverage of areas demonstrated to be strongly activated by optogenetic stimulation of CA1 using fMRI (Weitz et al., 2015). Our preparation yielded high quality chronic recordings of multiple interconnected areas, allowing us to assess the impact of optogenetic stimulation on the network. By varying the intensity of light stimulation, we measured ADTs using train stimuli (oADT), and the unitary PDT using single pulses (oPDT). We present the results of the oADT experiments first to provide context for the oPDT, but it should be noted that prior ADs, and/or exposure to behavioral seizures are not required to obtain oPDT measurements.

### Measuring the oADT and opto-kindling

**Figure 2.**
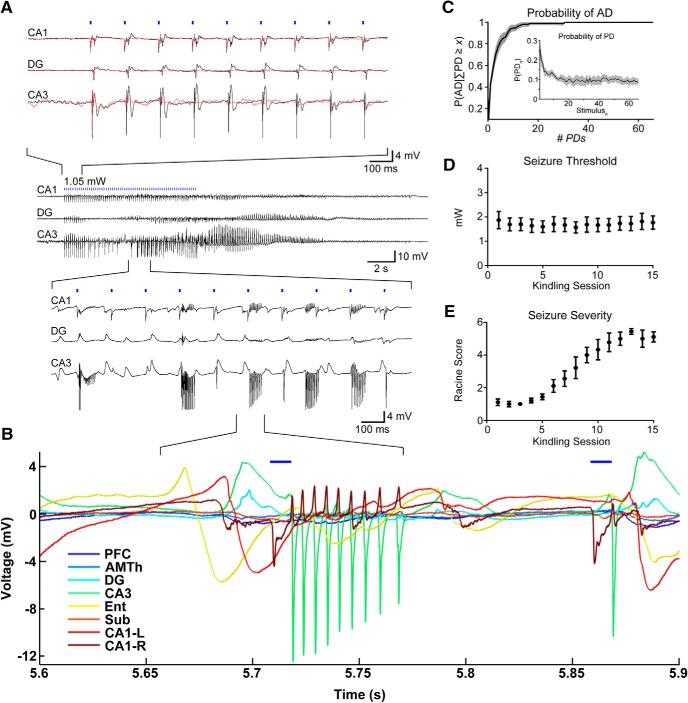
Optogenetic train stimulation produces an AD. Kindling with daily oADs results in an overt behavioral seizure. ***A***, Representative example of an optically-evoked AD. Top traces, Suprathreshold stimulation levels (for AD) produce network wide PDs (black traces). At subthreshold intensity levels, activation was observed only at the stimulation site (CA1-R; red traces). Middle traces, Zoomed out view of oAD. Bottom traces, pHFOs during oAD induction. ***B***, oADs involve pHFOs across multiple sites with a non-zero phase lag. ***C***, The probability of AD given at least × PDs occurring during the stimulus train. Inset, The overall probability of a PD on each pulse of the train, including subthreshold stimuli. Probabilities were calculated for each animal and the mean is displayed with 95% CIs, *n* = 7 animals. ***D***, Seizure threshold remained stable throughout the kindling period. ***E***, Behavioral seizure severity increased with each AD and was correlated with stimulation number.

oADs and behavioral seizures were induced using rhythmic square wave optical stimuli (6.67 Hz, 10-ms pulse, 10-s train); 6.67 Hz was the lowest frequency that reliably produced an oAD and the longer period (150 ms) provided sufficient time between pulses to observe the propagation of induced activity throughout the network (Fig. 2*B*). Stimulus trains were presented every 2 min at increasing light intensity until an oAD occurred (Fig. 2*A*,*C*). Subthreshold stimulation produced local activation at the stimulation site (CA1), but the evoked activity did not spread to downstream areas (Fig. 2*A*, top inset). The mean oADT was 2.11 mW (SD 1.12, 95% CI [1.08, 3.15], *n* = 7).

Our preparation is configured to stimulate CA1 with minimal damage by situating the optical fiber just above CA1 in visual cortex. In addition to strong expression in CA1, Thy1 mice also express ChR2 in layer 5 of cortex, raising the possibility that these cells were activated by light backscatter. To verify that CA1 is the relevant site of stimulation for seizure induction, we performed control experiments (*n* = 2) in which the fiber was placed superficially in cortex (0.5 mm dorsal to the fiber location in the standard array). Much higher light intensities were required to induce ADs in these animals (16.58 mW and 27.86 mW using a fiber coupled laser), levels sufficient to produce suprathreshold activation of CA1 from this distance, suggesting that activation of cortex is not sufficient to generate ADs and that CA1 is the primary site of activation.

While suprathreshold stimulation produced network wide oPDs following each pulse, high-frequency high-amplitude bursts, and oAD, occasionally the stimulation intensity just below threshold produced synchronous discharges similar to oPDs on the first few pulses (Fig. 2*A*, red traces, top). Likely a result of ChR2 desensitization (Lin, 2010), the probability of an oPD decreases with pulse number (Fig. 2*C*, inset), allowing us to estimate the number of oPDs required for an oAD. We found that the probability of oAD depends on the number of oPDs generated by the stimulus (Fig. 2*C*), P(AD) > 0.90 for *x* = 8 oPDs and P(AD)>0.99 for *x* = 17 oPDs (*n* = 841 stimulus trains). This suggests that the oAD is a result of the accumulated effect of closely spaced oPDs possibly related to the accumulation of extracellular potassium (de Curtis et al., 2018). oADs occurred during and immediately after stimulation (Fig. 2*A–C*), rather than after a delay, as previously observed with optogenetic seizure induction in motor cortex (Khoshkhoo et al., 2017). The mean oAD duration was 23.1 s (SD 4.84, 95% CIs [22.2, 24.0], *n* = 105 oADs).

Pathologic high-frequency oscillations (pHFOs; 80–500 Hz) have been detected preceding seizures in a number of animal models and in intracranial recordings from humans with epilepsy (Bragin et al., 1999; Staba et al., 2002; Zijlmans et al., 2012). In the majority (88%, *n* = 105 oADs) of optogenetically-induced oADs, pHFOs occurred in CA3, DG, and CA1 (Fig. 2*A*,*B*). A non-zero phase shift was observed between these areas, suggesting synaptic or ephaptic transmission, rather than volume conduction (Shivacharan et al., 2019; Fig. 2*B*). The mean latency from the start of the stimulation to burst onset was 6.42 s (SD 2.75, 95% CI [5.87, 6.97], *n* = 97 oADs). Epileptiform bursts ranged in frequency from 150 to 400 Hz (6.6- to 2.5-ms intraburst intervals) and were detected in all animals tested (7/7). These bursts were similar (in terms of amplitude and frequency) to patterns previously observed in perforant path-stimulated or kainate-treated rats (Bragin et al., 1997, 2004, 2005).

### Opto-kindling and the emergence of behavioral seizures

Using this thresholding procedure, we took a subset of animals (*n* = 7) through an opto-kindling procedure based on those performed previously with electrical stimulation (Goddard et al., 1969; Racine, 1972a; Fig. 2*D*,*E*). Threshold oADs were presented daily for 15 d. Traditional electrical kindling results in reduced thresholds and increased behavioral severity (Racine, 1972a). In contrast, the oADT remained stable (uncorrelated with presentation #) over the course of kindling (*r* = 0.0865, *p* = 0.759, ns, *n* = 7; Fig. 2*D*), in agreement with previously reported optogenetic seizure thresholds obtained by varying stimulus number (Khoshkhoo et al., 2017). The mean oADT during kindling was 1.99 mW (SD 0.827, 95% CI [1.22, 2.75], *n* = 7). Repeated oAD induction was correlated with increasingly severe behavioral seizures as quantified using a modified Racine scale (*r* = 0.963, *p* < 0.0001, *n* = 7; Fig. 2*E*). Animals progressed to stage five seizures after 9.6 d (SD 2.4, 95% CI [7.4, 12], *n* = 7), considerably faster than corneal or hippocampal kindled mice (Rowley and White, 2010; Stover et al., 2017), but slower than kindling of piriform cortex (McIntyre et al., 1993). The stereotypical behavioral expression (semiology) of the seizure was similar to that observed with electrical kindling (Racine et al., 1972; Kairiss et al., 1984). Spontaneous seizures were not observed during or following the 15-d kindling period.

### oPD induction and threshold measurement

Given the close relationship between the appearance of multiple network wide oPDs following stimulation and subsequent oADs, we sought to determine whether the oPD itself was a sufficient indicator of the oADT and therefore, a potential measure of oADT without the need for oAD induction.

**Figure 3.**
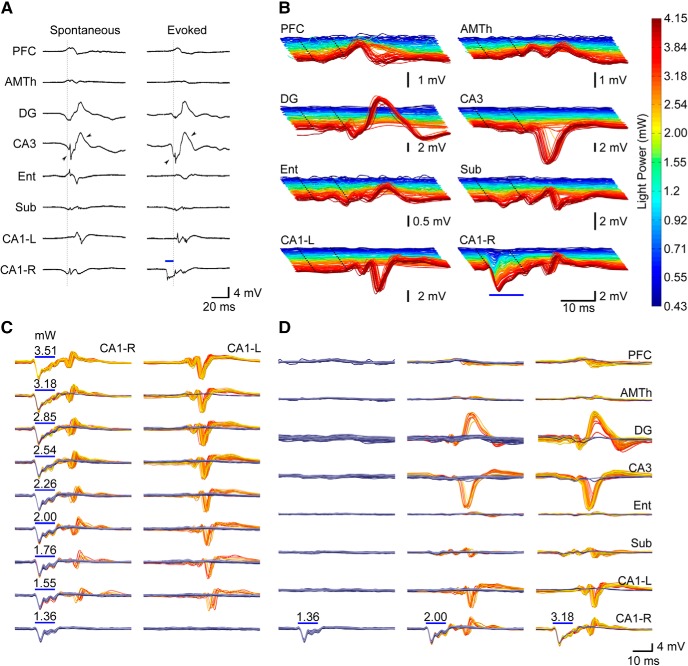
Optogenetic stimulation produces network wide PDs that closely resemble spontaneous IIS with a probability that increases with light intensity. ***A***, Example comparing spontaneous IIS to oPDs. Spontaneous IIS are characterized by near synchronous activation in DG, CA3, and CA1-R, and delayed activation in CA1-L. Evoked discharges are led by activation in CA1 (stimulation site) followed by near synchronous activation of DG, CA3, and CA1 with a latency of ∼10 ms. Note similar timing and wave shape between stimulus evoked and spontaneous discharges (arrowheads). Blue bars indicate onset and duration of light stimulus in CA1. ISI = 3 s. ***B***, Evoked network activity produced by optogenetic stimulation of area CA1 (right side) at varying light intensities in a representative animal (different from that depicted in ***A***). Note large amplitude oPDs evoked at higher intensities. Dotted lines indicate the beginning and end of the light pulse. ***C***, ***D***, Probability of oPD occurrence in downstream sites, rather than amplitude, increases with light intensity at the stimulation site. In area CA1-L, a longer latency response is accompanied by a high amplitude PD. The gold colored traces are those in which a PD was detected, the blue are traces where it was absent. Each plot is an overlay of 60 trials, and the color gradient indicates chronological order (dark blue/red for early trials, light blue/yellow for late trials). oPD occurrences: 0/60 at 1.36 mW, 11/60 at 2 mW, 58/60 at 3.18 mW. ISI = 3 s. Scale bars in ***D*** also apply to ***C***.

Single pulse optogenetic stimulation of CA1 at a sufficient intensity (mean 2.85 mW, SD 0.248, 95% CI [2.34- 3.36], *n* = 30 mice) produced nearly synchronous PDs across the hippocampal and perihippocampal network (Fig. 3*A*,*B*). The largest amplitude responses were observed in DG and CA3, as well as contralateral CA1. These unitary oPDs closely resembled those induced in the first few pulses of the optical stimulus train used in the oADT procedure, as well as spontaneous type-2 IIS, observed in AD-exposed animals (Fig. 3*A*).

To precisely measure the oPDT, we developed a novel optogenetic intensity-response curve procedure that takes advantage of the all or none transition between the sub and suprathreshold response at recording sites downstream of the optogenetic stimulation to accurately detect the occurrence of oPDs (Fig. 3). Repeated presentation of the intensity-response curve revealed considerable variability in the response (oPD vs no oPD) given a particular stimulus intensity (Fig. 3*C*,*D*). However, we found analyzing the probability of an oPD given stimulus intensity x, P(oPD|I_x_), allowed for a more robust estimation of threshold.

**Figure 4.**
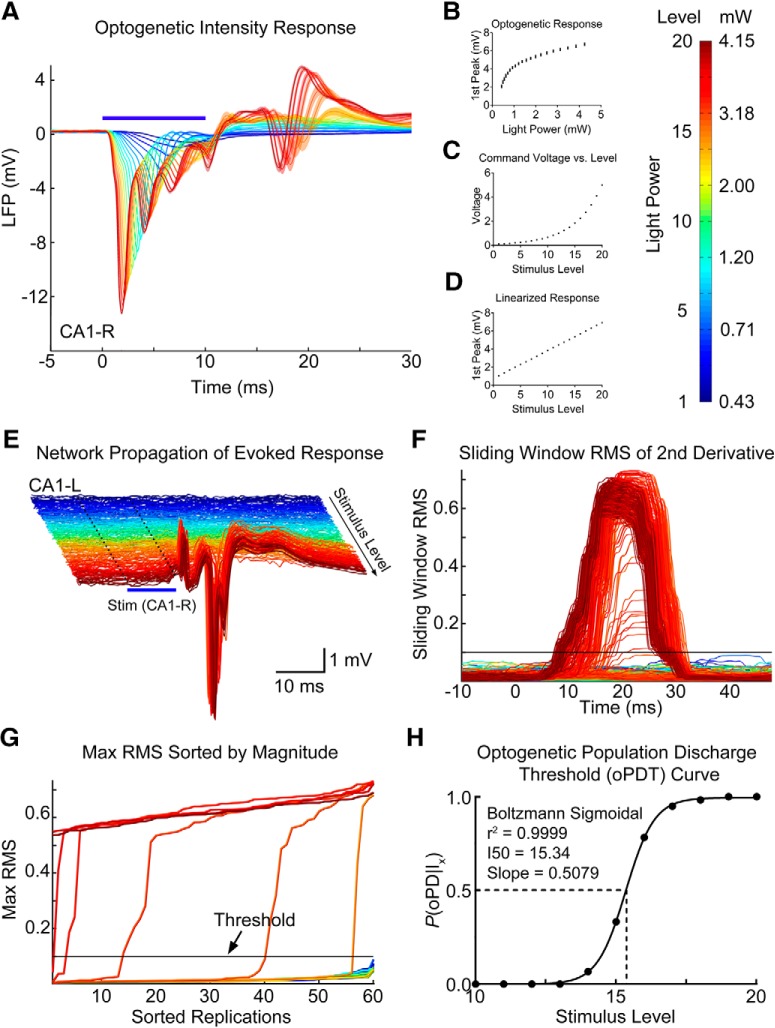
Summary of the oPDT procedure. ***A***, Average LFP response to optogenetic activation in CA1 at varying light intensities. Shaded bars = 95% CIs. ***B***, The short latency LFP response was used as an approximation of ChR2-mediated current to generate a light intensity-response curve. Mean and SD are displayed. ***C***, A series of light intensities was chosen to produce a linearized optogenetic response (***D***). ***E***, The network propagation of evoked activity from the stimulation site (CA1-R, blue bar) to CA1-L. Individual replicates plotted as bandpass filtered (5–300 Hz) traces. Colors correspond to the level of the light stimulus (shown in mW on color bar); 20 levels × 60 replicates = 1200 total stimuli. ISI = 3 s. Note high amplitude PDs. ***F***, Sliding window RMS of the 2nd derivative calculated from ***E***. Horizontal line indicates the threshold. ***G***, Max RMS (within time window) for each light level sorted by magnitude. Threshold is set in the steep part of the curve. ***H***, oPD ratio (oPDs/total stimuli) plot fit by the Boltzmann sigmoid allows for calculation of an I50, or the LED power at which 50% of the stimuli produce a oPD.

To generate the oPD probability curve we used twenty intensity levels presented in repeated blocks. Stimuli were presented in random order without replacement so that each stimulus intensity level was presented once per block. Stimulus order was independently randomized for each block. oPD probability was then estimated using multiple replications of stimulus blocks. The intensity levels were selected to linearize the optogenetic response as measured by the mean amplitude of the first peak at the stimulation site, likely a reflection of the interaction between the ChR2-mediated current and dendritic voltage-gated Na^+^ currents (Fig. 4*A–D*). Network oPDs were detected by thresholding a sliding window RMS of the 2nd derivative of the peri-stimulus activity (Fig. 4*E–G*).

The oPD occurs across the network, and therefore, any channel or combination of channels can be used for oPD detection, including the long latency response at the stimulation site; however, we found that contralateral CA1 provided the greatest separation between subthreshold and suprathreshold responses. The sub-supra separation was visualized by plotting the sorted peak values of the RMS for each level (Fig. 4*G*). Good separation produces a steep transition with few intermediate values. This plot is also useful for setting the threshold and the steepness of the transition reduces the detection bias introduced by threshold selection. As a result, the oPDT curve is robust to any choice of threshold within the steep transition region of the sorted peak RMS plot.

Once oPDs have been detected, the proportion of oPDs to total stimulus presentations at each level can then be plotted to generate an oPDT curve. We then fit the data with the Hodgkin and Huxley formulation of the Boltzmann distribution and calculated the I50, or the intensity at which the probability of oPD was 50% (Hodgkin and Huxley, 1952; Fig. 4*H*). Expressing the I50 in the arbitrary linearized scale (1–20 levels) is convenient for making comparisons, however, it can easily be converted into power (mW) or irradiance (mW/mm^2^; Fig. 4*B–D*). Although the linearized scale improves the fit of the Boltzmann, substituting mW also produces a sigmoidal curve. Using an arbitrary scale and baseline normalization allows for comparisons between animals even when the relationship between power output and optogenetic response varies. In our experience, the variability in baseline I50 between animals, SD = 3.41 levels (*n* = 20 animals), was larger than the mean I50 variability within animals, mean SD = 1.10 levels (*n* = 3–10 baselines per animal). Since ChR2 expression patterns are preserved between animals, it is likely that between-animal differences are due to variations in the surgical preparation and the transmission efficiency of the implanted fiber.

### Relationship between the oPDT and oADT

**Figure 5.**
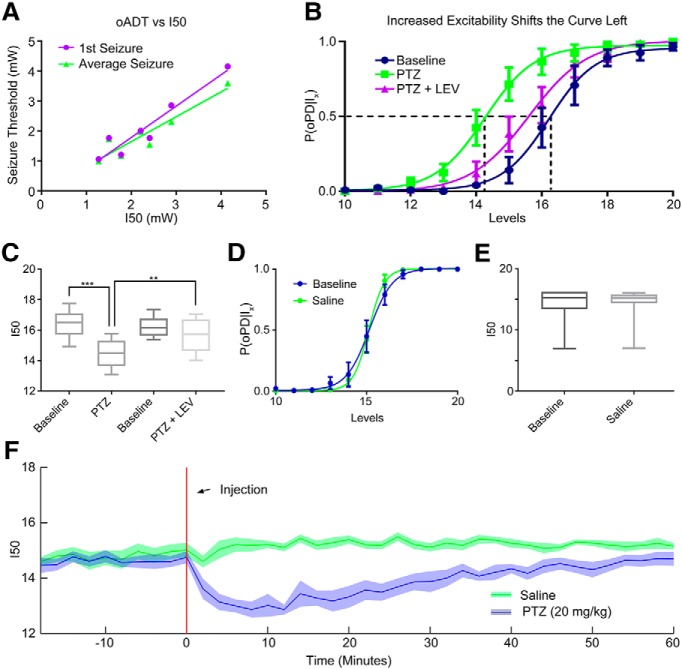
The I50 is sensitive to changes in network excitability. ***A***, The relationship between pre-seizure I50 and the 1st oADT (purple) or the average oADT throughout 15 kindling sessions (green). ***B***, ***C***, The GABAa antagonist PTZ (20 mg/kg) shifts the oPD curve leftward and treatment with the anticonvulsant drug LEV (40 mg/kg) partially reverses the shift; *n* = 6 animals; ***p = 0.00043, **p = 0.0034. Curve error bars indicate the 95% CI. ***D***, ***E***, Handling and intraperitoneal saline administration did not significantly shift the oPD curve compared to baseline (paired *t* test, *n* = 7). ***F***, I50 measurements reveal the time course of PTZ-induced hyperexcitability (disinhibition). Plot of the I50 over time. Six block bins, *n* = 9 presentations of each condition (three trials × three animals). Shaded area is mean ± 95% CI.

Our analysis of the oADT revealed that multiple oPDs must be evoked by the train stimulus to generate an oAD (Fig. 2*C*). We compared the oADT and the oPDT measured in the same animals using the same chronically implanted fiber. We found that the oADT was correlated with pre-kindling I50 across animals with a slope approaching 1 (1st seizure: lin. reg. slope: 1.05, 95% CI [0.677, 1.42], Pearson’s *r* = 0.956, *p* = 0.00078, *n* = 7; average seizure: lin. reg. slope: 0.830, 95% CI [0.462, 1.20], Pearson’s *r* = 0.933, *p* = 0.0021, *n* = 7; Fig. 5*A*). This finding combined with the fact that train stimuli subthreshold for the oPD also failed to produce an oAD, indicate that the oPDT and the oADT are equivalent in terms of light intensity delivered from the same implanted fiber, because both require the oPD.

### Changes in excitability shift the oPD curve

To test the hypothesis that the P(oPD|I_x_) changes with the balance of excitation and inhibition, we used a well-known chemoconvulsant drug, pentylenetetrazol (PTZ), in non-kindled animals. Despite its ability to induce acute seizures, PTZ does not produce a shift in the electrical ADT (Karler et al., 1989). In contrast, the oPDT was sensitive to PTZ at sub-convulsive doses (20 mg/kg). The I50 was significantly shifted to the left relative to baseline (baseline = 16.4 levels, SD 0.943, 95% CI [15.4, 17.4], PTZ = 14.5 levels, SD 0.949, 95% CI [13.5, 15.5], *p* = 0.00041, *n* = 6; Fig. 5*A*,*B*), which indicated a reduction in threshold.

Although the antiepileptic drug levetiracetam (LEV) failed to show efficacy in the traditional MES and subcutaneous PTZ acute seizure models (Löscher, 2011), it does increase the ADT in kindled rats (Löscher et al., 2000). Pre-treatment with LEV (40 mg/kg) partially reversed the leftward shift produced by subthreshold PTZ and returned threshold to near baseline levels (PTZ+LEV = 15.7 levels, SD 1.11, 95% CI [14.5, 16.8]; baseline vs PTZ, *p* = 0.00043, baseline vs PTZ+LEV, *p* = 0.12, PTZ vs PTZ+LEV, *p* = 0.0034, *n* = 6 repeated-measures ANOVA with Tukey’s correction for multiple comparisons; Fig. 5*A*,*B*). Saline injection did not significantly shift the curve (baseline vs saline, *p* = 0.99, *n* = 7, paired *t* test).

By breaking the stimulus period into bins of presentation blocks, P(oPD|I_x_) can be measured over time. Administration of subthreshold PTZ (intraperitoneally) produced a rapid shift in excitability that partially recovered by ∼60 min after injection (Fig. 5*F*), compared to saline, consistent with the pharmacokinetic curve of PTZ (Yonekawa et al., 1980). For this example, six block bins were used to estimate the I50 at 2-min intervals [six blocks × 20 stimuli per block × 1-s interstimulus interval (ISI) = 120 s), but higher temporal resolution is possible by decreasing the bin size and/or the number of stimuli per block.

### Stability and reliability of the oPDT

We assessed the reliability of oPDT measurements, in terms of short- and long-term stability, by analyzing baseline data. Two ISIs (1 and 3 s) were used to produce intensity-response curves. This allowed for a comparison of the stability of baseline I50 values generated using each protocol, with the caveat that the data came from different animals. Baselines were selected for further analysis if they were collected in naïve animals (pre-kindling, pre-treatment; *n* = 67 sessions in 13 animals for 1-s, *n* = 41 sessions in 17 animals for 3-s interval).

**Figure 6.**
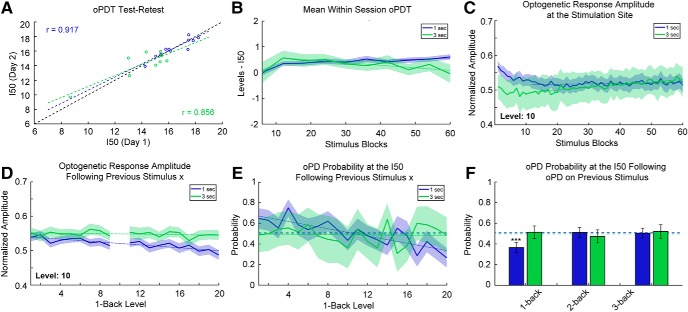
Stability and reliability of the oPDT. ***A***, Test-retest comparison of session I50s on consecutive days; *n* = 11 pairs for 1-s interval and *n* = 9 pairs for 3-s interval. Each session I50 is calculated from 60 replicates. Dashed black line indicates the diagonal. ***B***, I50 is relatively stable over the course of a session. Mean normalized intermediate I50s (six replicate bins) for 1- and 3-s ISIs. Each stimulus block consisted of 20 intensity levels presented in random order. Order was randomized independently for each block. Session duration is 20 min for the 1-s ISI and 60 min for the 3-s ISI; *n* = 67 and *n* = 41 sessions for the 1- and 3-s ISIs, respectively. Shaded bars indicate 95% CI. Dotted lines indicate best fit (linear regression) excluding blocks 1–12. ***C***, Optogenetic response amplitude is reduced within the first 5 min of recording, likely due to ChR2 desensitization. Values were transformed via min-max normalization for the entire curve. Same dataset as in ***B***. Dotted lines indicate best fit (linear regression) excluding blocks 1–12. ***D***, The optogenetic response amplitude at the stimulation site is reduced following high intensity stimuli (1-back stimuli are plotted on the *x*-axis). The *y*-axis indicates the mean normalized response amplitude at level 10. Same dataset as in ***B***. ***E***, The probability of an oPD at the I50 depends on the intensity of the previous stimulus for 1-s intervals. Dashed lines indicate the overall probability of an oPD at a level within ±0.25 of the I50. Dotted lines indicate best fit (linear regression); 1 s: *n* = 29 sessions, 1740 trials, 3 s: *n* = 10 sessions, 600 trials. ***F***, oPDs that occur <2 s prior have a suppressive effect on the probability of an oPD at the I50. Dashed lines indicate the overall probability of an oPD at a level within ±0.25 of the I50. Error bars indicate the 95% CI. Same subset as in ***E***.

First, we looked at the test-retest reliability of measurements (20 levels, 60 replications each) taken on consecutive days (Fig. 6*A*). The measured I50s were consistently reliable with an overall correlation coefficient > 0.85 (1-s interval: *r* = 0.917, 95% CI [0.705, 0.979], *p* < 0.0001, *n* = 11 pairs, 3-s interval: *r* = 0.856, 95% CI [0.446, 0.969], *p* = 0.0032, *n* = 9 pairs, Pearson’s).

Mono-synaptic stimulation of CA1 afferents at 1-s intervals has been shown to induce long-term depression (LTD) *in vivo* (Thiels et al., 1994; Oliet et al., 1997; Massey and Bashir, 2007). To determine whether the multi-synaptic I50 was drifting over time in our preparation, we broke each 60-block session into 10× six-block bins and calculated intermediate I50 values normalized by subtracting the overall session I50 (Fig. 6*B*). Following a transient increase for both, session I50 values gradually increased for the 1-s interval and decreased for 3-s interval (1-s interval: slope greater than zero, slope: 0.00442 [0.00206, 0.00678], *p* = 0.0031, 3-s interval: slope less than zero slope: –0.0101 [–0.0158, –0.00441], *p* = 0.0040, linear regression of the means excluding the first 12 replicates).

This gradual shift in the I50 through time could be a result of plasticity in the network but also ChR2 desensitization at the stimulation site. All ChR variants exhibit desensitization with varying degrees of reduction in peak photocurrents following repeated activation (Lin, 2010; Williams et al., 2013). We checked for ChR2 desensitization by measuring the peak amplitude (session min/max normalized) of the optogenetic response at the stimulation site over time (Fig. 6*C*). We found that the response amplitude is reduced (3%) within the first 4 min, likely an effect of desensitization, after which it increased slightly (1-s interval: slope greater than zero, slope: 1.16 × 10^−4^, 95% CI [7.62 × 10^−6^, 2.25 × 10^−4^], *p* = 0.037, 3-s interval: slope greater than zero, slope: 7.63 × 10^−4^, 95% CI [6.52 × 10^−4^, 8.75 × 10^−4^], *p* < 0.0001, linear regression of the means excluding the first 12 replicates). Overall, the baseline drift in the response amplitude and the I50 over time was small relative to the effects of subthreshold PTZ, for instance (Fig. 5*B*,*F*). Nevertheless, drift must be accounted for in the interpretation of experimental results and all results should be compared to a vehicle control (Fig. 5*E*,*F*).

Next, we examined possible short-term effects of stimulation on the P(oPD|I_x_) and assessed trial independence. The extent of ChR2 desensitization depends on the strength of previous activation and higher intensity stimuli are expected to produce greater desensitization than lower intensity stimuli. Since the stimuli are presented in random order, we were able to quantify this effect by measuring the extent of desensitization produced by each stimulus pair. We measured the peak amplitude of the response to a stimulus (level 10) following each of the other stimulus levels (Fig. 6*D*). There was a small reduction in amplitude of the response for strong preceding stimuli (1-s interval: slope less than zero, slope: –0.00208, 95% CI [–0.00271, –0.00145], *p* < 0.0001, 3-s interval: slope not different from zero, slope: –3.58 × 10^−4^, 95% CI [–9.56 × 10^−4^, 2.40 × 10^−4^] *p* = 0.22, ns, linear regression of the means). to determine whether this change in amplitude translated into a shift in the oPD probability, we calculated the oPDT probability at or near the I50 for each preceding stimulus (Fig. 6*E*). A subset of the data was selected for this analysis restricted to those sessions with I50 values that were near a presented stimulus level (within ± 0.25 levels of a whole number; 1 s: *n* = 29 sessions, 1740 trials, 3 s: *n* = 10 sessions, 600 trials). This allowed us to determine whether deviations from chance were related to the stimulus order. The probability of oPD was negatively associated with the intensity of the previous stimulus for the 1-s but not 3-s interval (1-s interval: slope less than zero, slope: –0.0177, 95% CI [–0.0232, –0.0121], *p* < 0.0001, 3-s interval: slope not different from zero, slope: –8.37 × 10^−4^ 95% CI [–0.008, 0.007], *p* = 0.82). This suggests that the reduction in amplitude (Fig. 6*D*) translates into a reduction in oPD probability.

Higher intensity stimuli are also more likely to produce an oPD, so we checked for effects of the oPD itself on the oPD probability at the I50. We calculated the conditional probability of an oPD at stimulus levels within ±0.25 of the I50 (where the probability of an oPD should be close to chance), given an oPD on the nth previous stimulus (any level). Each *n*-back probability was calculated independently. We found a strong suppressive effect of a previous PD for the 1-back stimulus with a 1-s ISI compared to the overall probability of an oPD at the I50 (*p* < 0.0001, Fisher’s exact test, corrected for multiple comparisons using the Holm–Sidak method). PD probability did not differ significantly from chance at 2- and 3-back for the 1-s interval (*p* = 0.91 and *p* = 0.32, respectively, Fisher’s exact test, corrected). PD probability remained at chance for 1-, 2-, and 3-back for the 3-s interval (*p* = 0.91, *p* = 0.51, and *p* = 0.45, respectively, Fisher’s exact test, corrected). Therefore, an interval of >2 s is expected to be sufficient to produce independent trials and the 1-s interval is expected to produce I50s that slightly overestimate the oPDT, likely due to a combination of ChR2 desensitization and after-hyperpolarization produced by the oPD. For this reason, it is important to compare measurements to baselines collected using the same protocol with the same ISI. It is important to note that all of the above measurements were taken from awake behaving animals and include random variation due to changes in ongoing activity. Taken together, these results indicate that the oPDT curve offers exceptional precision and reliability and as such provides a new clear window into the excitability state of the intact brain.

## Discussion

Neuronal activity occurs on a background of synaptic and intrinsic excitability governed by the biophysical properties of the component cells, synaptic plasticity, and the control of these parameters by internal homeostasis and brainstem modulatory systems (Sterling and Laughlin, 2015). The complex dynamics of neural circuitry makes it difficult to predict excitability from discrete measurements in reduced preparations, and obtaining direct measurements in behaving animals is technically challenging (Petersen, 2017). Changes in excitability relevant to seizure susceptibility are not fully captured by measurements of individual contributing factors and determining how these factors should be combined to define network excitability is a non-trivial problem. Using a novel light intensity-response procedure, we have developed and validated a new quantitative estimate of network excitability state based on PD probability. Network PDs are an unambiguous indicator of the transition from normal to abnormal functional states; by mapping the probability distribution of oPDs as a function of input magnitude, we derive a surrogate measure of network excitability, the I50, that depends not only on the excitability of the population of cells directly stimulated, but also on the receiving cells in the rest of the network. This approach has several important features: first, combining optogenetic modulators with a high precision LED light source allows for tighter control over induced currents in specific cell populations than previously possible with electrical stimulation; second, our multi-site recording technique allows us to simultaneously induce and monitor PDs as they propagate throughout the network, critical for unambiguous classification; third, our chronic preparation permits tracking of excitability dynamics over multiple timescales, from minutes to months; and finally, because the oPDT procedure does not produce seizures and the baseline I50 is relatively stable over time, pharmacological or molecular interventions can be tested within subjects, greatly increasing the ability to detect functional changes as a result of genetic and/or pharmacological manipulation.

The electrical ADT has been used extensively as a measure of seizure threshold (Löscher, 2017). In many ways, the oADT is the optogenetic equivalent of the electrical ADT, both produce similar acute ADs and repeated presentations lead to behavioral seizure (kindling). Although seizures are an unmistakable sign that a threshold has been crossed, they also produce long lasting effects on the brain, including widespread changes in gene expression (Altar et al., 2004). In contrast, the oPDT does not produce seizures, nor kindling, and therefore can be used to measure thresholds over time allowing within-subjects comparisons free from the confounds of seizure exposure. This property may allow the oPDT to be used to track changes in excitability in models of epileptogenesis as a way to gain insight into mechanisms and measure the effectiveness of various pharmacological and molecular interventions.

Previously optogenetic seizure thresholds have been determined by varying the number of stimulus bouts (Khoshkhoo et al., 2017). The oADT represents the first example of systematic optogenetic intensity-response curves, using a high-precision LED, to precisely determine seizure thresholds (keeping stimulation frequency, pulse width, and train duration constant). When the oADT and the oPDT are combined, the oADT, in the context of a known oPDT provides additional information about seizure susceptibility. Although the thresholds for both metrics are the same in terms of light intensity, the oADT requires repeated oPD inductions to produce an AD. This suggests that distinct mechanisms are involved in the oAD generation, possibly the accumulation of extracellular K^+^ and the switch to depolarizing GABA (Cossart et al., 2005; Miles et al., 2012; Alfonsa et al., 2015; Buchin et al., 2016). Comparing the results of each approach might allow for identification of drugs and manipulations that prevent AD but do not change baseline excitability, and therefore can be expected to have fewer side effects.

Electrically-evoked potential amplitude has previously been used as a measure of excitability in animal models and patients with epilepsy (Maru and Goddard, 1987; Freestone et al., 2011; Enatsu et al., 2012; Wendling et al., 2016; Keller et al., 2018). However, without knowing the details of underlying synaptic currents and their influence on the LFP, it is difficult to estimate excitability from amplitude alone given that somatic inhibition and dendritic excitation produce similar sink/source patterns (Buzsáki et al., 2012; Hales and Pockett, 2014; Herreras, 2016). Because network wide PDs are readily differentiated from subthreshold synaptic potentials, our preparation provides an unambiguous measurement of excitability that does not require knowledge of the sink/source patterns needed to interpret the LFP itself.

Electrical stimulation activates cells and fibers of passage which act on a sparse and widely distributed population of neurons whose identity cannot be predicted ahead of time, nor known with certainty afterward (Histed et al., 2009). In contrast, optogenetic expression patterns can readily be determined histologically and light spread can be accurately modeled based on fiber location and power output. Light spreads out in a cone from the tip of the fiber and drops off quickly with distance in brain tissue due to scattering (Aravanis et al., 2007; Yizhar et al., 2011). As the light intensity increases, cells directly under the fiber will be increasingly depolarized to threshold followed by neighboring cells. Thus, the optogenetic intensity-response curve varies both the magnitude of depolarization in individual cells as well as the overall size of the activated population. Cells in the center that have already reached threshold cannot be depolarized further, in part because ChR2 currents are voltage dependent (Williams et al., 2013). From the perspective of downstream areas, the intensity-response curve varies the number of synchronously active projections emanating from the stimulation site. This is important because, while synchronous firing is an important feature of the function of these circuits, exceeding normal ranges would be expected to produce non-specific propagation of the synchronous discharge when some critical number of downstream cells is induced to fire synchronously.

The sharp transition between sub and suprathreshold activity may reflect a breakdown between the balance of feedforward inhibition and excitation (Buzsáki, 1984; Wahlstrom-Helgren and Klyachko, 2016). If the magnitude of the population EPSP exceeds that of feedforward inhibition, it could produce a highly synchronous discharge that would propagate to other areas (Johnston and Brown, 1981). This would explain the sensitivity of the oPDT curve to GABA antagonists such as PTZ. In addition to the balance of inhibition and excitation, a number of other functional properties might be expected to modulate the oPDT, the oADT, or both. Synaptic strength determines the threshold for downstream propagation of the synchronous discharge (Nicoll, 2017), intrinsic excitability modulates the responsiveness of downstream cells, which is governed by the relative expression and cellular localization of voltage-gated Na^+^, K^+^, and Ca^2+^ channels (Graef and Godwin, 2010; Meadows et al., 2016; Lisman et al., 2018), and the regulation of the extracellular space by glia (Devinsky et al., 2013).

The Thy1-ChR2 mice (line 18) used in these experiments express high levels of wild-type ChR2-EYFP in deep (calbindin-negative) pyramidal neurons in CA1 (Arenkiel et al., 2007; Dobbins et al., 2018). Importantly, transgenic animals provide more consistent and specific ChR2 expression patterns than could be achieved with virus injection. Compared to neighboring superficial cells, deep pyramidal neurons in CA1 are more prone to burst firing, have unique input and output pathways, and lack calcium buffering proteins, potentially making them more susceptible to seizure activity (Sloviter, 1989; Mizuseki et al., 2011; Valero et al., 2015). By selectively targeting this population of excitatory neurons the inhibitory population is free to respond, preserving the strong reciprocal relationship they have with the excitatory cells being stimulated, and allowing them to participate in the generation of the synchronous discharge (Cobb et al., 1995; Ellender et al., 2014; Sessolo et al., 2015; Yekhlef et al., 2015; Khoshkhoo et al., 2017; Wang et al., 2017; Chang et al., 2018a; Magloire et al., 2019).

The fact that the evoked discharges initiated in CA1 occurred simultaneously in multiple hippocampal structures supports the hypothesis that synchronous activation of Ent was responsible (Yeckel and Berger, 1990; Avoli et al., 2002). The oPD was most prominent in areas CA3 and DG; reciprocally connected areas prone to hypersynchronous discharge (Traub and Jefferys, 1994). CA3 and DG lead during spontaneous discharges suggesting that synchronous inputs to these areas are responsible for generating the IIS, and optogenetically generated synchronous input from CA1 via Sub and Ent serves as a trigger for the oPD. These results are also consistent with the dentate gate hypothesis, which posits that dentate granule cells, because of their relatively high functional threshold, act as a gate that prevents the propagation of seizure activity into the rest of hippocampus (Heinemann et al., 1992; Lothman et al., 1992; Goldberg and Coulter, 2013). Although hippocampus has multiple interacting nested loops, if the DG is the last to reach “critical mass,” a synchronous discharge there could be responsible for the all-or-none expression of the oPD. It this is true, the oPDT, in its current configuration, is a sensitive measure of the strength of the “dentate gate” and could be useful for evaluating therapeutic interventions in this area (Krook-Magnuson et al., 2013, 2015). Non-synaptic propagation, often neglected in earlier studies of seizure, is also likely to play a role in generating the synchronous discharge in the closely situated structures of the hippocampus (Zhang et al., 2014).

Despite the widespread synchronous nature of the discharge, it is transient, and the system is surprisingly resilient to its effects on short time scales. By analyzing the oPD probabilities from one stimulus to the next, we found that the response is functionally independent of the previous stimulus in just a few seconds. Furthermore, closely spaced oPDs (1-s ISI) are suppressive to the system, significantly reducing the probability of another oPD in response to the subsequent stimulus. The suppressive effect of the oPD is likely due to a combination of ChR2 desensitization and the slow after-hyperpolarization (I_sAHP_; Alger and Nicoll, 1980; Lancaster and Adams, 1986). The fact that unitary PDs are suppressive and multiple repeated oPDs are required to initiate seizure suggests that either there is a reduction in the suppressive effect with each oPD, or that multiple PDs produce sufficient excitation to overcome the suppressive effects. In the context of spontaneous seizure, this makes it clear that seizures are a result of a slow transition to abnormal brain states, such as those presumably induced by persistence repetitive synchronous discharges in our model, which are permissive to self-sustaining seizure activity and that they are not simply the result of transient synchronous activity alone (Chang et al., 2018b).

The oPDT procedure allows for multiple threshold measurements over time; an important advantage over previous methods. For comparison, the recovery time for the 6-hz psychomotor seizure and minimal electroshock threshold is at least 4.4 and 7.5 min, respectively (Brown et al., 1953). The enhanced temporal resolution of the oPDT can be used to estimate the functional pharmacokinetics of anti-epileptic drugs or to study the natural fluctuations in excitability that depend on brain state and behavior (Baud et al., 2018, 2019). The temporal resolution of the oPDT is limited by the number of stimuli used to generate the curve and the ISI. As we have demonstrated, stimuli are functionally independent when presented with ISI > 2 s. Further reductions could be achieved by using a reduced subset of stimuli, optimized for baseline curves in each animal. For small changes, sampling at the I50 alone should be sufficient. When implemented in a closed-loop system, optimization techniques could be used to further reduce the number of stimuli necessary (MacKay, 1992).

On longer time scales (days to weeks), repeated stimulation of the brain is expected to produce a number of changes, some of which involve changes in plasticity and homeostatic regulation of the stimulated cells (Thiels et al., 1994; Oliet et al., 1997; Massey and Bashir, 2007). Recently, it was demonstrated that long-term (24 h) optogenetic stimulation of CA1 produces changes in spine density and synaptic plasticity (Moulin et al., 2019). Although we have demonstrated that the oPDT is stable over time, appropriate control experiments should be used and care should be taken when interpreting results to account for potential changes in the brain as a result of stimulation.

The current study is focused on using optogenetic intensity-response curves to quantify excitability in the context of seizure activity, but the same procedure could be applied to any study that uses optogenetic stimulation as the independent variable with a stochastic dependent variable. Similar procedures could be performed with any appropriate optogenetically-induced cellular, molecular, physiologic, or behavioral output metric (Tischer and Weiner, 2014; Allen et al., 2015; Bugaj et al., 2016). Abnormal excitability is also associated with a number of other neurologic disorders including depression, autism, Alzheimer’s, anxiety, drug addiction (and withdrawal), and schizophrenia (Santos et al., 2010; Friedman et al., 2014; Kourrich et al., 2015; Rau et al., 2015; Crabtree et al., 2017; Takarae and Sweeney, 2017). Correlating changes in excitability with behavior or neurochemical state in the context of these disorders may provide novel insights into the mechanisms of the underlying disease.

In summary, we show that synchronous optogenetic activation is sufficient to induce epileptiform PDs, and the systematic modulation of light intensity allows for precise estimation of the threshold between normal and abnormal activity. This approach provides a novel platform for testing the effects of therapeutic interventions on network excitability.

10.1523/ENEURO.0229-18.2019.ed1 Extended data 1 Supplementary optoDR - example output. Download Extended data 1, ZIP file.

10.1523/ENEURO.0229-18.2019.ed2 Extended data 2 Supplementary optoDR - complete example data file. Download Extended data 2, ZIP file.

10.1523/ENEURO.0229-18.2019.ed3 Extended data 3 Supplementary optoDR code - no data. Download Extended data 3, ZIP file.

## Synthesis

Reviewing Editor: William Stacey, University of Michigan Decisions are customarily a result of the Reviewing Editor and the peer reviewers coming together and discussing their recommendations until a consensus is reached. When revisions are invited, a fact-based synthesis statement explaining their decision and outlining what is needed to prepare a revision will be listed below. The following reviewer(s) agreed to reveal their identity: Andrew Trevelyan, Kevin Staley.

We have discussed your submission and find that the work is well-written and interesting, potentially suitable for publication in eNeuro. However, there are several issues that need to be addressed.

1. The most critical concern is that it is necessary to show why this new method with optogenetic manipulation is different/better than the “old standard” of afterdischarge threshold. There are several sources that have used ADC to measure excitability (e.g. pubmed numbers 17116389,

2322833 and 19399874). Is this just a low intensity version of ADC, using optogenetics? Also, as described by Reviewer 2, there are some concerns about the conclusions made for assessing this oEDT50, and the reviewer offers several suggestions of how to show some differences between these two methods (How does the oEDT50 change compared to the ADC over the course of kindling? How does the ADC change in the presence of PTZ and levetiracetam, as in figure 4? How is oEDT50 different? Better?).

2. There should be a better description of how the stimuli were optimized and how robust the responses are, as described by Rev 1.

3. The analysis of Fig. 5f raises some concerns and should be clarified (Rev. 1 comments).

In addition, Rev. 1 has included many minor textual comments that should be addressed.

Reviewer 1:

This in vivo study develops an interesting optogenetic assay of network excitability, that is then used to assess seizure susceptibility. The main methodology is monitoring the postsynaptic discharges following a focal optogenetic activation of CA1 pyramidal cells. They also trigger seizures the same way, referring to this as optogenetic kindling. It is not entirely novel, because others have done similar things (eg Ellender et al, examining the output of PV cells during epileptiform discharges in vitro; and Khoshkhoo et al, 2017, triggering seizures optogenetically, in neocortex). The electrophysiological readout was at multiple locations in both ipsi- and contralateral hippocampal and parahippocampal territories. They developed an intuitive experimental protocol, nicely illustrated (although note my comments below about the text descriptions, which were at times somewhat confusing), and which served to normalise the datasets from different animals, leading to quite precise assessments of altered seizure susceptibility. For instance, they were able to show a nicely delineated time profile for the effect of a low dose PTZ injection. They also showed a very good correlation with epileptic kindling efficacy, delivered optogenetically from the same optrode. Having said that, the seizures are being triggered by the same optrode as is used for the assay, so this is perhaps not that surprising a result. I think this should be flagged up in the discussion (see comments below).

Broadly speaking, I believe the results, and I thought that the experimental protocol was clever, and is likely to be of great interest to the field. The text, however, was a little muddled at times, with some rather ambiguous statements and descriptions, and somewhat imprecise on occasions. My comments below are largely aimed at improving the descriptions of the study. I consider these mostly to be relatively minor comments. They are listed in the order they appear in the MS.

Line 44: seizure should be plural (“underlie seizures themselves”)

Line 47 and multiple other instances: “superthreshold” used interchangeably with “suprathreshold” - I think the latter, but whichever, it should be consistent.

Ln 51: “Frohlich” in capitals - also in the Refs (line 427)

Ln 71 & 74: Author University / Author Funding source / Author publication

Line 82: “optotrode” - optrode?

Line 102: “where” should be “were”

Results: The presentation of results rather leapt back and forth in a way that was quite confusing at first - eg suddenly introducing “Seizure exposed animals (Figure 4)”, straight after Figure 2, and without any statement of how the seizures arose, and then not talking about this line of experiments at all for another couple of pages.

Line 162: “Suggesting the discharge is initiated downstream” - referring to the latency of 10ms between optogenetic stimulation and the discharge. That doesn't seem too long to me. A more convincing case would be if they showed multiunit activity (MUA) at another location prior to the CA1 MUA.

Line 163: “bistable” - I think “binary” is more accurate

Line 167: should be “in an ED”

Figure 2b - It would help the reader I think, if these traces were presented as a single column of panels, without the slanted offset in each block, to permit an easier assessment of the relative timing of discharges at the various sites. Having said that, I realise that these essentially repeat what is shown in (a).

Figure 3 legend: (f) “Vertical lines” - where are these? (g) Threshold is set just above the inflection point - please be more precise.

In figure 3 itself - (g) Can “Reps” be written “repetitions”; (h) oPD ratio - should be oED? Also, I'm not quite clear what the ratio is - was this stated somewhere?

The descriptions of the analysis in figure 3 could be made more clear, especially with regard to how a rather extensive set of stimuli (taking up to an hour), can be fine tuned such that the sampling of the key parameter space is optimised (ie limiting the illumination strategies to the steep parts of the cumulative frequency plots - fig 3g). For starters, it might help to demonstrate that the entire analysis is robust to any choice of threshold in the steep part of the curves - it would just give a shifted I50.

Lines 189-91: I wasn't entirely clear how the CoV was calculated, or what was being averaged (line 190, “means”)

Lines p197f: Assessment of non-stationarity - The important thing here is not so much the reduced variance, but rather, evidence of progressive changes, is successive groups of trials. The authors acknowledge that 1s repeats are too frequent, but I suspect the 3s repeats may also show drift (the CoV analysis is not sensitive to this). This is important, because it is an added incentive for optimising the stimulation strategy.

Line 205: please give PTZ dose

Figure 4 - should the saline data be shown also in the boxplots?

Line 224: “modified kindling procedure (Goddard et al, 1969; Racine, 2972b) - this is referring I believe to optogenetic kindling, but referencing these old studies made me misread this initially as implying that the kindling is electrical. Assuming it is indeed optogenetic, then these refs should be omitted here, but put elsewhere. The Khoshkhoo reference, which also uses optogenetic kindling, albeit in neocortex, might be more appropriate.

Figure 5 - Might the CA1/DG/CA3 labels be put on the other traces in a - might be helped also by color-coding. Also, why not show the other locations. An analysis of the high frequency component would also help illuminate where the seizure actually takes root. It looks like it starts in CA3, but my money would be on the entorhinal cortex, which was recorded but not shown. Please show, and comment.

Fig 5f - this correlation at first seems impressive, but less so once one realises that it is essentially a correlation between two different effects downstream of the two different illumination strategies of the same optrode. This point rather reduces the impact of what might otherwise be a dramatic result, and perhaps explains why it was slightly brushed under the carpet. It does need to be made explicit, I feel, because the future utility of this experimental approach will be to assess seizure susceptibility arising from other sources and by other mechanisms. Instead, the only discussion of this point (line 321-3: ”suggests that propagation probability is not the deciding factor that differentiates spikes from seizures“) is too cryptic, and seems to go beyond what I would conclude, given my point above.

Line 243: ”Epileptiform bursts ranged in frequency from 150-400Hz“ is ambiguous. They are not happening at that frequency, although they may have a component of that - but even so, it will still be dwarfed by the power in the lower bands. The description just needs to be more precise.

Line 394: No volume / page numbers

The Endnote settings includes unnecessary detail: viz.

Lines 391 & 412: J. Neurosci. Off. J. Soc. Neurosci -

Line 500: Alcohol Alcohol. Oxf. Oxfs.

Reviewer 2:

This is a well-written and illustrated report of a thoughtfully executed study of an optical measure of excitability, the oEDT50, in optically kindled rhodopsin-expressing animals.

The central issue is how much more information is provided by the oEDT50 vs. the afterdischarge threshold (ADT). The ADT is a measure that has long been used to describe the minimum required electrophysiological stimulus intensity required for electrical kindling, and it is also used in this study. How does the oEDT50 change compared to the ADC over the course of kindling? How does the ADC change in the presence of PTZ and levetiracetam, as in figure 4?

In Figure 4 the authors show that the oEDT 50 is altered by subconvulsant doses of PTZ conclude that the oEDT50 is a useful parameter for describing the seizure threshold (line 256). I don't think this has been proven by the experiments in figure 4. Rather, these experiments show that the oEDT50 correlate well with disinhibition; without seizures , it is not clear that the oEDT50 correlates with seizure threshold except as induced at the same site by optical kindling.

The equation of the oEDT50 with spontaneous interictal spikes needs to be qualified - the spontaneous spikes occur as a consequence of kindling at the site at which the oEDT50 is also elicited. As a thought experiment, do the authors think that the oEDT50 would trigger discharges that were identical to spontaneous interictal spikes that were recorded following kainate or pilocarpine-induced status epilepticus?

It would be helpful to discuss the oEDT50 in terms of what has been learned using the afterdischarge threshold. How is oEDT50 different? Better?

## Author Response

Reviewer Comments:

We thank the reviewers for their helpful comments, useful insights, and great ideas for future experiments. We have made all of the suggested changes and believe the manuscript is better as a result.

Two notes on nomenclature:

1) In naming the experimental approach we developed, we sought to use a term that was both novel, descriptive, and without restrictive associations to any specific clinical phenomena. Clinically, epileptiform discharge can refer to spikes or poly-spikes, of varying duration. ED is also shorthand for ”effective dose“, and is commonly used in the characterization of anti-convulsants. Spikes are a commonly used term to describe EDs, but also have very different definitions when viewed from the clinical vs. neuroscience perspective. Furthermore, the inter vs ictal designation is not useful when the discharges are being experimentally induced. Therefore, after much deliberation, we have decided to use the more general term ”population discharge“ to name the technique; optogenetic population discharge threshold (oPDT).

2) We use the term “intensity” to refer to a quantity of light per unit area as is common in optogenetic studies, rather than the more specific radiometric definition commonly used in astronomy (W/sr). In order to standardize light levels between labs, we report light values in power (mW) at the fiber tip. For those interested in the gradient of irradiance (mW/mm2) values across the three dimensional volume of stimulated tissue, we suggest this helpful conversion tool: https://web.stanford.edu/group/dlab/cgi-bin/graph/chart.php. We use the term “intensity response curve” to refer to the arbitrary scale developed to account for the complex transformation between light power at the fiber tip and the optogenetic response in expressing cells.

Synthesis of Reviews:

Computational Neuroscience Model Code Accessibility Comments for Author (Required):

Please include this section under a separate heading “Code Accessibility”. And please establish the github with the link as described.

Significance Statement Comments for Author (Required):

n/a

Comments on the Visual Abstract for Author (Required):

n/a

Synthesis Statement for Author (Required):

We have discussed your submission and find that the work is well-written and interesting, potentially suitable for publication in eNeuro. However, there are several issues that need to be addressed.

1. The most critical concern is that it is necessary to show why this new method with optogenetic manipulation is different/better than the “old standard” of afterdischarge threshold. There are several sources that have used ADC to measure excitability (e.g. pubmed numbers 17116389,

2322833 and 19399874). Is this just a low intensity version of ADC, using optogenetics? Also, as described by Reviewer 2, there are some concerns about the conclusions made for assessing this oEDT50, and the reviewer offers several suggestions of how to show some differences between these two methods (How does the oEDT50 change compared to the ADC over the course of kindling? How does the ADC change in the presence of PTZ and levetiracetam, as in figure 4? How is oEDT50 different? Better?).

We agree that an expanded discussion of the ADT and related methods would help to place the oPDT in context. We have reframed the introduction and referenced the ADT (lines 74-77 of the revised manuscript), and substantially revised the discussion (lines 417-448 of the revised manuscript) to compare and contrast the new techniques (oADT and oPDT) with current and historical standards. Additional changes related to specific reviewed critiques are discussed below.

2. There should be a better description of how the stimuli were optimized and how robust the responses are, as described by Rev 1.

We have added a detailed characterization of the reliability and stability (short and long-term) of the oPDT (lines 320-377 of the revised manuscript), along with a new figure depicting these results (fig. 6).

3. The analysis of Fig. 5f raises some concerns and should be clarified (Rev. 1 comments).

We now emphasize the fact that figure 5f (Fig. 5a in the revised manuscript) represents oPD and oAD thresholds collected from the same animals using the same stimulation fiber (lines 294-302). Although the oPDT and the oADT are equivalent in terms of light intensity (not a surprising result as the reviewers point out), we now make it clear that multiple oPDs are required to produce an AD. Furthermore, we now quantify this relationship by estimating how many oPDs are required to produce an oAD (line 221-229 and fig. 2c). We have also added additional discussion of the relationship between the oPDT and the oADT (lines 417-434) and suggest that they are complimentary metrics that depend on distinct but inter-related phenomena.

In addition, Rev. 1 has included many minor textual comments that should be addressed.

Thank you for the detailed review, we have made the recommended changes.

Reviewer 1:

This in vivo study develops an interesting optogenetic assay of network excitability, that is then used to assess seizure susceptibility. The main methodology is monitoring the postsynaptic discharges following a focal optogenetic activation of CA1 pyramidal cells. They also trigger seizures the same way, referring to this as optogenetic kindling. It is not entirely novel, because others have done similar things (eg Ellender et al, examining the output of PV cells during epileptiform discharges in vitro; and Khoshkhoo et al, 2017, triggering seizures optogenetically, in neocortex).

The opto-seizure results, while not entirely novel from the standpoint of the literature were developed independently in our lab and are unique in their implementation. Khoshkhoo 2017 performed “threshold” measurements using stimulus number but did not systematically vary light intensity. Instead, they varied frequency and intensity for each experiment as needed to achieve seizures, likely a limitation of the laser system the used for stimulation. Both of the mentioned references (Ellender and Khoshkhoo) were included in the original manuscript as were Osawa 2013 and Weitz 2015 which are closer in implementation to what we describe here but did not include optical intensity-response curves. We also note that our first descriptions of optokindling and seizure were in 2014 in abstract form.

The electrophysiological readout was at multiple locations in both ipsi- and contralateral hippocampal and parahippocampal territories. They developed an intuitive experimental protocol, nicely illustrated (although note my comments below about the text descriptions, which were at times somewhat confusing), and which served to normalise the datasets from different animals, leading to quite precise assessments of altered seizure susceptibility. For instance, they were able to show a nicely delineated time profile for the effect of a low dose PTZ injection. They also showed a very good correlation with epileptic kindling efficacy, delivered optogenetically from the same optrode. Having said that, the seizures are being triggered by the same optrode as is used for the assay, so this is perhaps not that surprising a result. Correct! I think this should be flagged up in the discussion (see comments below).

We regret any confusion about the details of this result (the near equivalence of the oPDT and the oADT in terms of light intensity) and its interpretation. We have made several changes to address this (detailed below).

Broadly speaking, I believe the results, and I thought that the experimental protocol was clever, and is likely to be of great interest to the field. The text, however, was a little muddled at times, with some rather ambiguous statements and descriptions, and somewhat imprecise on occasions. My comments below are largely aimed at improving the descriptions of the study. I consider these mostly to be relatively minor comments. They are listed in the order they appear in the MS.

Line 44: seizure should be plural (“underlie seizures themselves”) - fixed

Line 47 and multiple other instances: “superthreshold” used interchangeably with “suprathreshold” - I think the latter, but whichever, it should be consistent. - changed all instances to “suprathreshold”

Ln 51: “Frohlich” in capitals - also in the Refs (line 427) - fixed

Ln 71 & 74: Author University / Author Funding source / Author publication - these were for the purposes of blinding, I have replaced them.

Line 82: “optotrode” - optrode? - yes, fixed

Line 102: “where” should be “were” - fixed

Results: The presentation of results rather leapt back and forth in a way that was quite confusing at first - eg suddenly introducing “Seizure exposed animals (Figure 4)”, straight after Figure 2, and without any statement of how the seizures arose, and then not talking about this line of experiments at all for another couple of pages.

We agree that the original order was confusing. I have changed the order so that the oADT and kindling comes first, followed by the oPDT. I have moved figure 5f (now figure 2) to figure 5a.

The two metrics (oADT and oPDT) are inter-related and it is difficult to describe one without referencing the other. Although the new order fixes the issues described above, it also requires a brief mention of the oPD (lines 213-220) in the section on the oADT with a note pointing to reader to the oPDT figure (line 210). We find that presenting the PD first in relation to its role in AD generation helps the reader to understand what we are referring to when we use the term PD, as well as its relationship with the AD and seizure.

Line 162: “Suggesting the discharge is initiated downstream” - referring to the latency of 10ms between optogenetic stimulation and the discharge. That doesn't seem too long to me. A more convincing case would be if they showed multiunit activity (MUA) at another location prior to the CA1 MUA.

Upon further reflection, the term “discharge” is not sufficiently clear to describe the causal relationship between the interacting areas that participated. If discharge is meant to indicate cell firing, then CA1, the area of stimulation, is the site of the discharge. The population spike in CA1, as a result of direct optogenetic stimulation, is linearly related to the intensity of the light (by design). The downstream discharge (which we have termed PD) is a probabilistic high amplitude all or none event that occurs across the network and shows up as a lagged response on the CA1 electrode well after the light pulse has terminated. I have re-worded this section (lines 259-263) and eliminated the phrase “downstream discharge”.

Line 163: “bistable” - I think “binary” is more accurate

We agree, we also considered the word “bifurcation” because I think that term more accurately describes the situation than bistable (which implies two stable states and I am not sure that the PD could be considered a stable state). A bifurcation refers to a rapid change in the system from one state to another without making any judgement about stability. Binary does the same. Ultimately, however, we decided to go with the jargon-free “all or none” (line 265).

Line 167: should be “in an ED” - fixed (entire section was revised).

Figure 2b - It would help the reader I think, if these traces were presented as a single column of panels, without the slanted offset in each block, to permit an easier assessment of the relative timing of discharges at the various sites. Having said that, I realise that these essentially repeat what is shown in (a). Also shown in c and d, with PDs colored yellow-red (Fig. 3).

Figure 3 legend: (f) “Vertical lines” - where are these? My mistake, the vertical lines were included in a previous version of the figure. I have removed it from the legend.

(g) Threshold is set just above the inflection point - please be more precise. As discussed below, the threshold is not critical as long as it is set in the steep part of the max RMS curve. I removed the inflection point statement (lines 281-283).

In figure 3 itself - (g) Can “Reps” be written “repetitions”; Fixed

(h) oPD ratio - should be oED? Fixed. Also, see note about nomenclature above.

Also, I'm not quite clear what the ratio is - was this stated somewhere?

It is the ratio of evoked PDs to total stimuli (probability). I have clarified in the legend (fig 4h), and there is a short description is in the accompanying text (lines 284-285).

The descriptions of the analysis in figure 3 could be made more clear, especially with regard to how a rather extensive set of stimuli (taking up to an hour), can be fine tuned such that the sampling of the key parameter space is optimised (ie limiting the illumination strategies to the steep parts of the cumulative frequency plots - fig 3g). For starters, it might help to demonstrate that the entire analysis is robust to any choice of threshold in the steep part of the curves - it would just give a shifted I50.

We do routinely use a limited version of the curve (10 levels around the I50) after we determine baseline I50s using the full curve. Intermediate I50s can be calculated from much fewer replicates as well, as depicted in figure 4c (now 5f). As few as 6 replicates produce a reasonable estimate of the I50 with only 1 min of stimulation (10 levels and 1 sec intervals). I have added a detailed analysis of ISI (Fig. 6), and suggest additional possibilities for optimization in the discussion (lines 489-494).

I have also clarified the description to indicate that the threshold can be set within a wide range over the steep part of the curves (lines 281-283).

Lines 189-91: I wasn't entirely clear how the CoV was calculated, or what was being averaged (line 190, “means”).

The COV, CV, or RSD is a standard measure commonly used in analytical sciences as a measure of the repeatability of an assay. It is the ratio of the standard deviation to the mean expressed as a percentage and can be through of as “normalized” SD. However, upon further reflection, we have decided that because there is no reason to expect the variance to change with the mean, it is not necessary to use the CV. We have replaced this metric with the SD (lines 290-293).

Lines p197f: Assessment of non-stationarity - The important thing here is not so much the reduced variance, but rather, evidence of progressive changes, is successive groups of trials. The authors acknowledge that 1s repeats are too frequent, but I suspect the 3s repeats may also show drift (the CoV analysis is not sensitive to this). This is important, because it is an added incentive for optimising the stimulation strategy.

We agree that stability and trial independence are critical for building confidence in the oPDT as a metric of network excitability. We have performed a detailed assessment of short and long term stability of the oPDT and added an additional figure with the results of that analysis (Fig 6. and lines 320-377).

Line 205: please give PTZ dose - fixed

Figure 4 - should the saline data be shown also in the boxplots?

I have added a boxplot for the saline data (fig. 5e).

Line 224: “modified kindling procedure (Goddard et al, 1969; Racine, 2972b) - this is referring I believe to optogenetic kindling, but referencing these old studies made me misread this initially as implying that the kindling is electrical. Assuming it is indeed optogenetic, then these refs should be omitted here, but put elsewhere. The Khoshkhoo reference, which also uses optogenetic kindling, albeit in neocortex, might be more appropriate.

I have made changes throughout the manuscript to make it clear that the kindling is optogenetic kindling. I have also changed all instances of ”seizure threshold“ to ”oADT“, to further clarify. Although Khoshkhoo et. al. use optogenetic seizure induction, they did not use a kindling procedure (daily threshold ADs until behavioral seizure emerges), as far as I can tell. I kept the references to the first kindling experiments, but added the qualifier ”based on those performed previously with electrical stimulation“ (lines 243-244).

Figure 5 - Might the CA1/DG/CA3 labels be put on the other traces in a - might be helped also by color-coding. Also, why not show the other locations. An analysis of the high frequency component would also help illuminate where the seizure actually takes root. It looks like it starts in CA3, but my money would be on the entorhinal cortex, which was recorded but not shown. Please show, and comment.

I have added additional labels as well as a color coded overlay of all channels to figure 5 (now fig. 2). Entorhinal activation does in fact lead the pHFO, when the seizure becomes free running (no longer time locked to the optogenetic stimulus) (Fig. 2b). The dynamic interactions between the stimuli and seizure activity are complex and depend on the relative timing of each. A detailed analysis of these interactions is planned for a future publication.

Fig 5f - this correlation at first seems impressive, but less so once one realises that it is essentially a correlation between two different effects downstream of the two different illumination strategies of the same optrode. This point rather reduces the impact of what might otherwise be a dramatic result, and perhaps explains why it was slightly brushed under the carpet. It does need to be made explicit, I feel, because the future utility of this experimental approach will be to assess seizure susceptibility arising from other sources and by other mechanisms. Instead, the only discussion of this point (line 321-3: ”suggests that propagation probability is not the deciding factor that differentiates spikes from seizures“) is too cryptic, and seems to go beyond what I would conclude, given my point above.

Our point with this comparison was simply that the oPDT and oADT are essentially the same, in terms of light intensity. This should not be taken to mean that the oPDT and the oADT are equivalent phenomena. The oADT requires multiple oPDs in order to generate an AD (we have now quantified this relationship, fig. 2c). Importantly, ADs cannot be induced with train stimuli that are subthreshold for the oPD. In order to avoid confusion, we have clarified these points throughout the text (lines 221-229, 239-241, 294-302).

Line 243: ”Epileptiform bursts ranged in frequency from 150-400Hz“ is ambiguous. They are not happening at that frequency, although they may have a component of that - but even so, it will still be dwarfed by the power in the lower bands. The description just needs to be more precise.

They are actually occurring at that frequency as evidenced by the inter-burst period. See figure 5c (now 2c). The reviewer is correct that the envelope of the burst will contribute significant power in the lower bands, and the sharpness of the spikes will contribute higher harmonics. But the burst itself is occurring around 150 hz (5-10 ms inter-burst period). The 400Hz in the description was actually observed (inter-burst periods as short as 2.5 ms), and not a higher harmonic of a slower oscillation. An example is included below. The first 10 cycles (not counting the doublets) occur in ~25ms which comes out to ~400 hz, corresponding to the large peak at that frequency in the FFT. I have further clarified in the text (line 225).

Line 394: No volume / page numbers Fixed

The Endnote settings includes unnecessary detail: viz.

Lines 391 & 412: J. Neurosci. Off. J. Soc. Neurosci

Line 500: Alcohol Alcohol. Oxf. Oxfs. These citations were not included in the revised version.

Reviewer 2:

This is a well-written and illustrated report of a thoughtfully executed study of an optical measure of excitability, the oEDT50, in optically kindled rhodopsin-expressing animals.

The central issue is how much more information is provided by the oEDT50 vs. the afterdischarge threshold (ADT). The ADT is a measure that has long been used to describe the minimum required electrophysiological stimulus intensity required for electrical kindling, and it is also used in this study.

In the revised version, we have clarified the distinction between the oADT (fig. 2) and the oPDT (fig. 4). Both are presented in the paper, but they are fundamentally different and likely measure different things.

The oADT is very similar to the electrical ADT, but possesses all the advantages of optogenetic stimulation over electrical, namely cellular specificity and a lack of activation of fibers of passage. In the context of the current study, the greatest advantage of optogenetic stimulation is the ability to selectively target excitatory cells, leaving the inhibitory population free to respond. This preserves the timing relationship between these cell populations, which may be important for generating the synchronous discharge that underlies the oPD.

The oPDT is unique in that it measures the threshold for the unitary epileptiform discharge, without producing an AD. This property allows for multiple measurements over time and within subject comparisons. While the oPDT and the oADT are related (in terms of light intensity), the oADT requires multiple PDs (~8) in order to progress into AD. So while the oPD is sensitive to the excitability state of the system, the oADT further requires changes that occur on a longer timescale as a result of multiple oPDs, likely extracellular K+ accumulation and the switch to depolarizing GABA. These points have been highlighted in the discussion (lines 426-428).

To avoid confusion, we now indicate where optical methods were used with a lowercase ”o“. For example, oAD refers to an after-discharge that was induced by optical stimulation.

How does the oEDT50 change compared to the ADC over the course of kindling?

We now point out that the oADT remains stable over the course of kindling (as reported in the original manuscript) whereas electrical kindling tends to have a reduced threshold over the course of kindling (lines 245-248). Kindling also progressed faster with optical stimulation than has been reported with electrical stimulation (lines 250-253).

How does the ADC change in the presence of PTZ and levetiracetam, as in figure 4?

We now emphasis the inability of the electrical ADC to detect PTZ induced hyperexcitability, or the anticonvulsive properties of levetiracetam in contrast to the oPDT procedure (lines 304-314).

In Figure 4 the authors show that the oEDT 50 is altered by subconvulsant doses of PTZ conclude that the oEDT50 is a useful parameter for describing the seizure threshold (line 256). I don't think this has been proven by the experiments in figure 4. Rather, these experiments show that the oEDT50 correlate well with disinhibition; without seizures, it is not clear that the oEDT50 correlates with seizure threshold except as induced at the same site by optical kindling.

I have made it clear that I am referring to the oADT specifically rather than seizure generally (line 385). Furthermore, the paper has been re-organized to emphasize the complimentary nature of the oPDT and the oADT. Although both have the same threshold in terms of light intensity, the oADT additionally requires multiple oPDs to occur which suggests a change in the system over a longer time scale (10s or so). By comparing the two, one can separate out those manipulations that change excitability (such as disinhibition) vs. those that effect the transition to oAD.

The equation of the oEDT50 with spontaneous interictal spikes needs to be qualified - the spontaneous spikes occur as a consequence of kindling at the site at which the oEDT50 is also elicited. As a thought experiment, do the authors think that the oEDT50 would trigger discharges that were identical to spontaneous interictal spikes that were recorded following kainate or pilocarpine-induced status epilepticus?

This is a fantastic question and the subject of an ongoing study. Opto-kindling does seems to pattern subsequent spontaneous activity, even adopting certain features of the stimulus evoked response. We are very excited about these results and that is probably all I should say about it for now, stay tuned!

It would be helpful to discuss the oEDT50 in terms of what has been learned using the afterdischarge threshold. How is oEDT50 different? Better?

As described above, I have expanded the discussion of these points. Thanks!
