# Supplementary figures and images for: Optogenetically-Induced Population Discharge Threshold as a Sensitive Measure of Network Excitability
Source: eNeuro. 2019 Nov 6;6(6):ENEURO.0229-18.2019. doi: 10.1523/ENEURO.0229-18.2019 (PMC6838688; doi:10.1523/ENEURO.0229-18.2019)

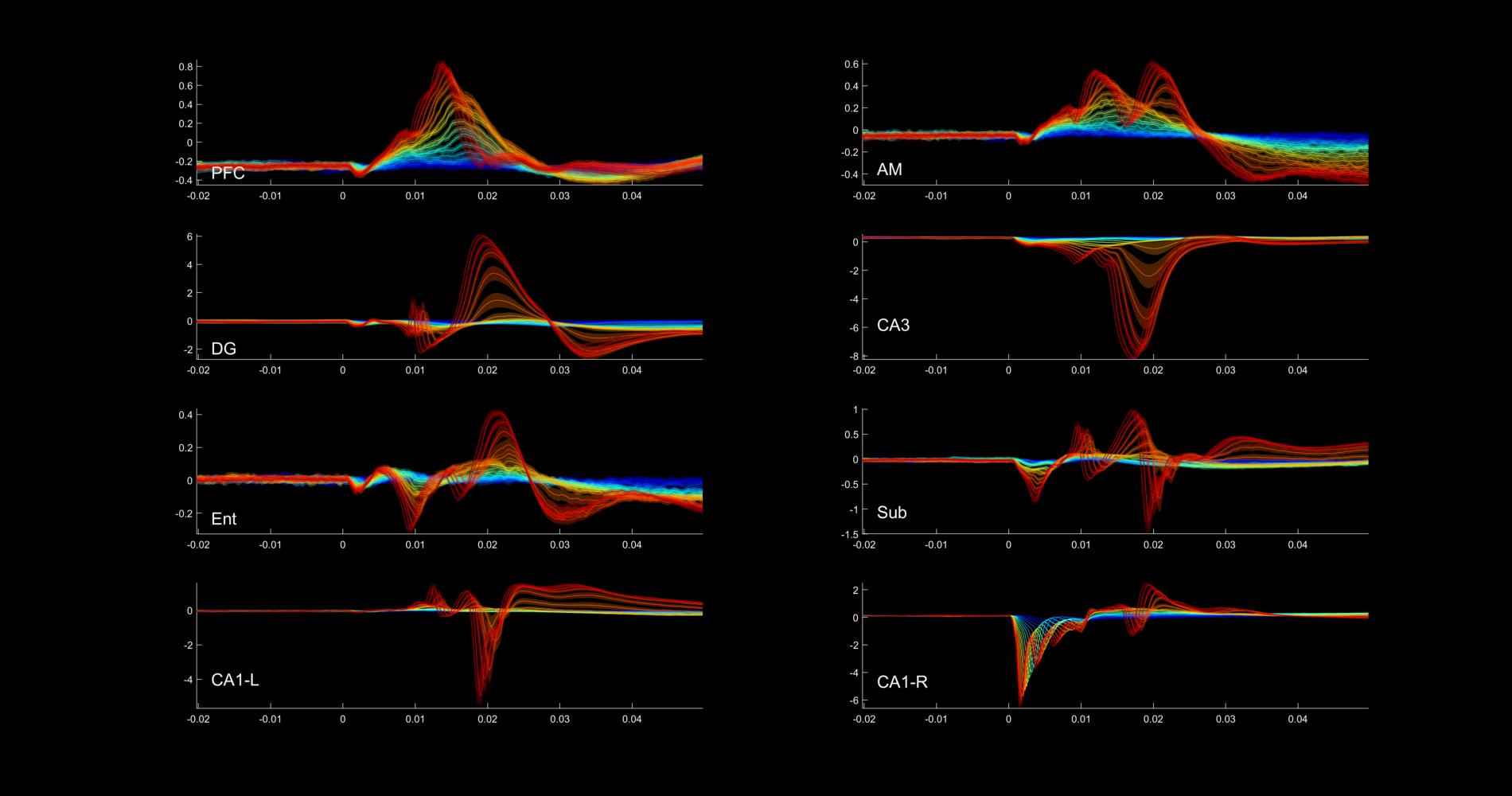

Supplement: Extended data 1 — Supplementary optoDR - example output. Download Extended data 1, ZIP file. [file sup_enu-eN-MNT-0229-18-s01.zip › SID_11-25-15_0003_all_ci.png]

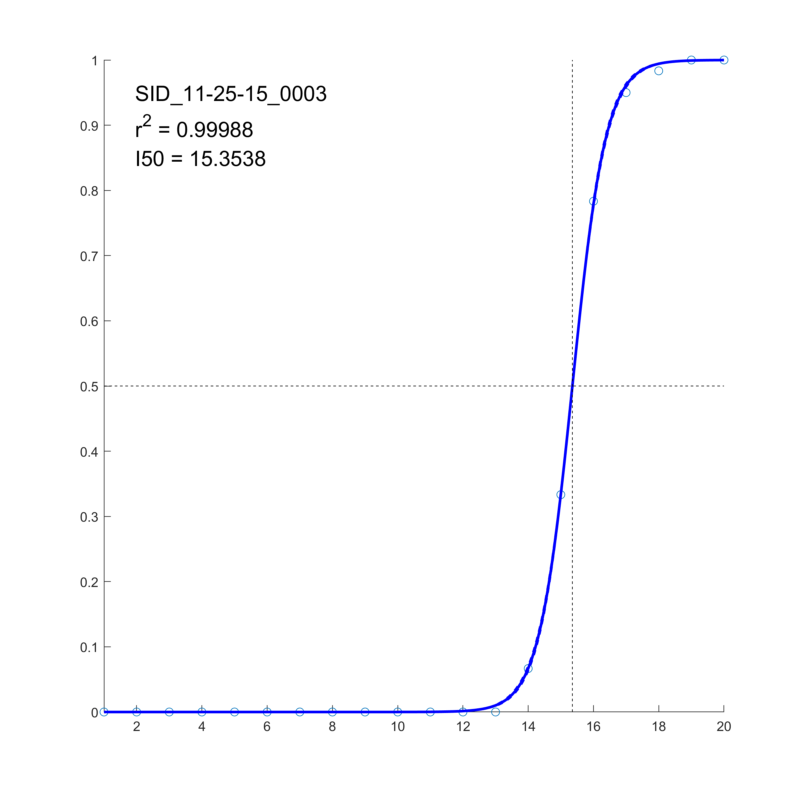

Supplement: Extended data 1 — Supplementary optoDR - example output. Download Extended data 1, ZIP file. [file sup_enu-eN-MNT-0229-18-s01.zip › SID_11-25-15_0003_curves_summary_60_reps.png]

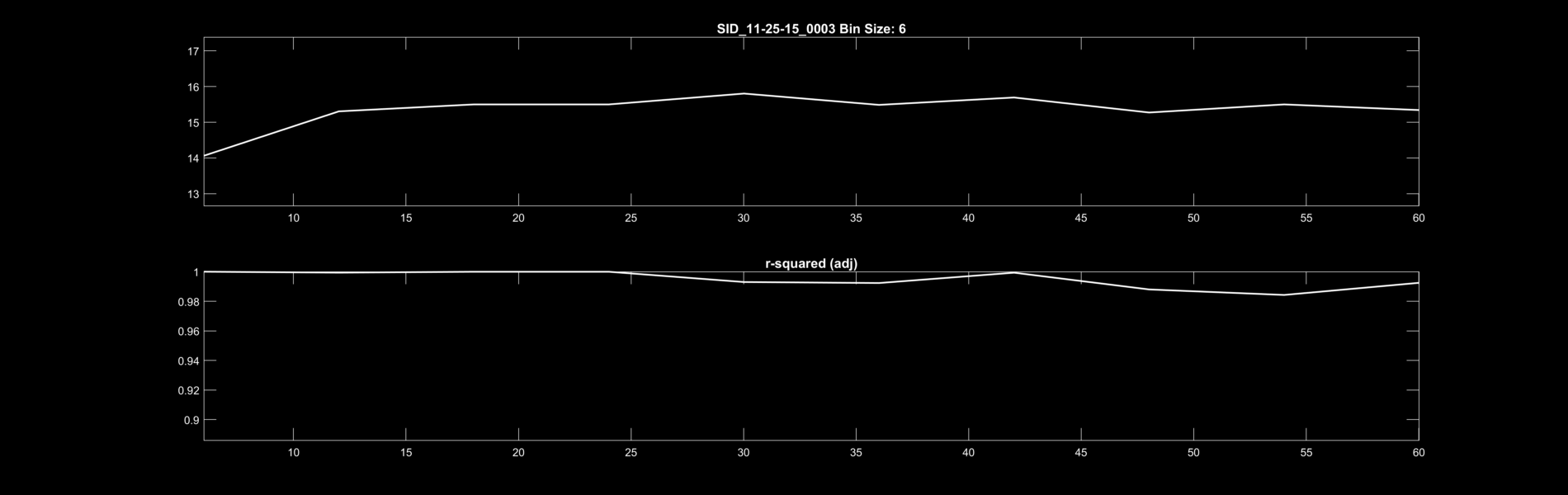

Supplement: Extended data 1 — Supplementary optoDR - example output. Download Extended data 1, ZIP file. [file sup_enu-eN-MNT-0229-18-s01.zip › SID_11-25-15_0003_I50_Bin_6.png]

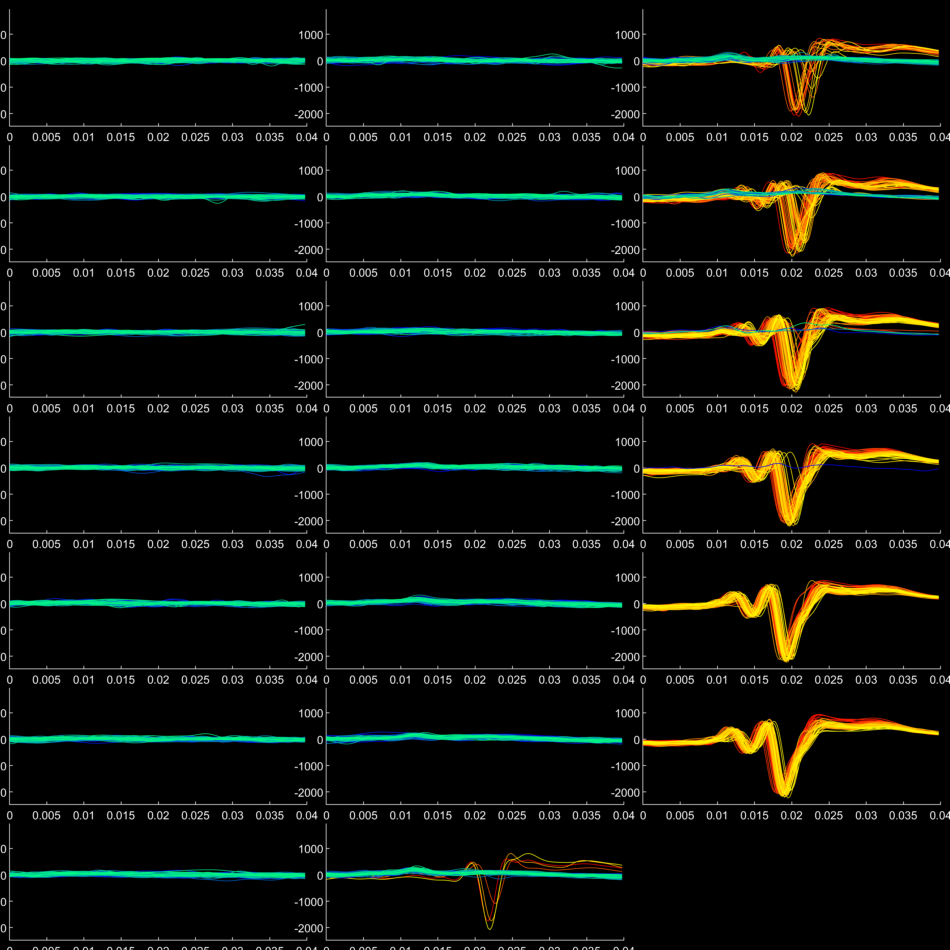

Supplement: Extended data 1 — Supplementary optoDR - example output. Download Extended data 1, ZIP file. [file sup_enu-eN-MNT-0229-18-s01.zip › SID_11-25-15_0003_PS_sep_plot_ch7_60_reps.png]

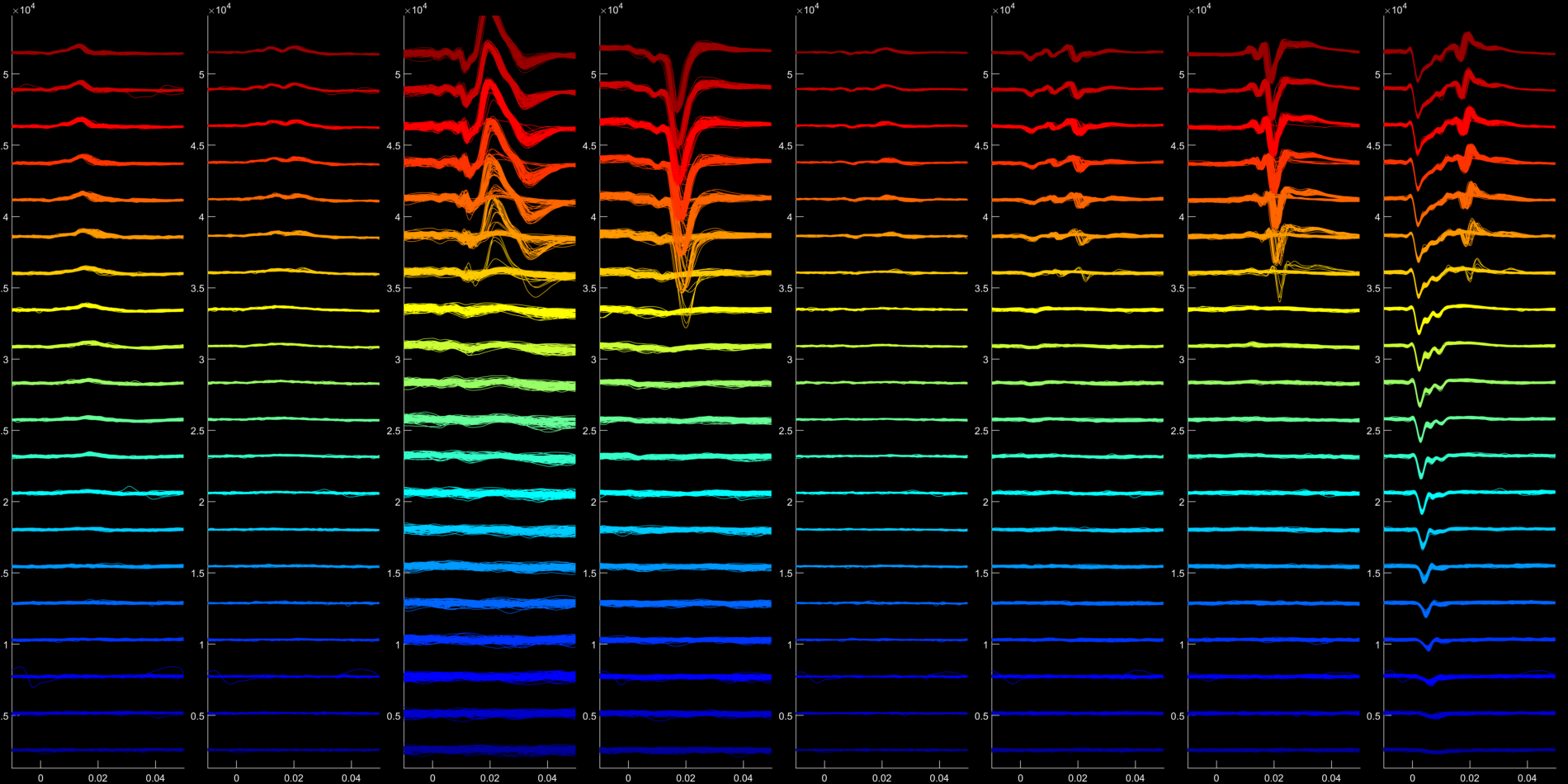

Supplement: Extended data 1 — Supplementary optoDR - example output. Download Extended data 1, ZIP file. [file sup_enu-eN-MNT-0229-18-s01.zip › SID_11-25-15_0003_raw_filt_all_60_reps.png]

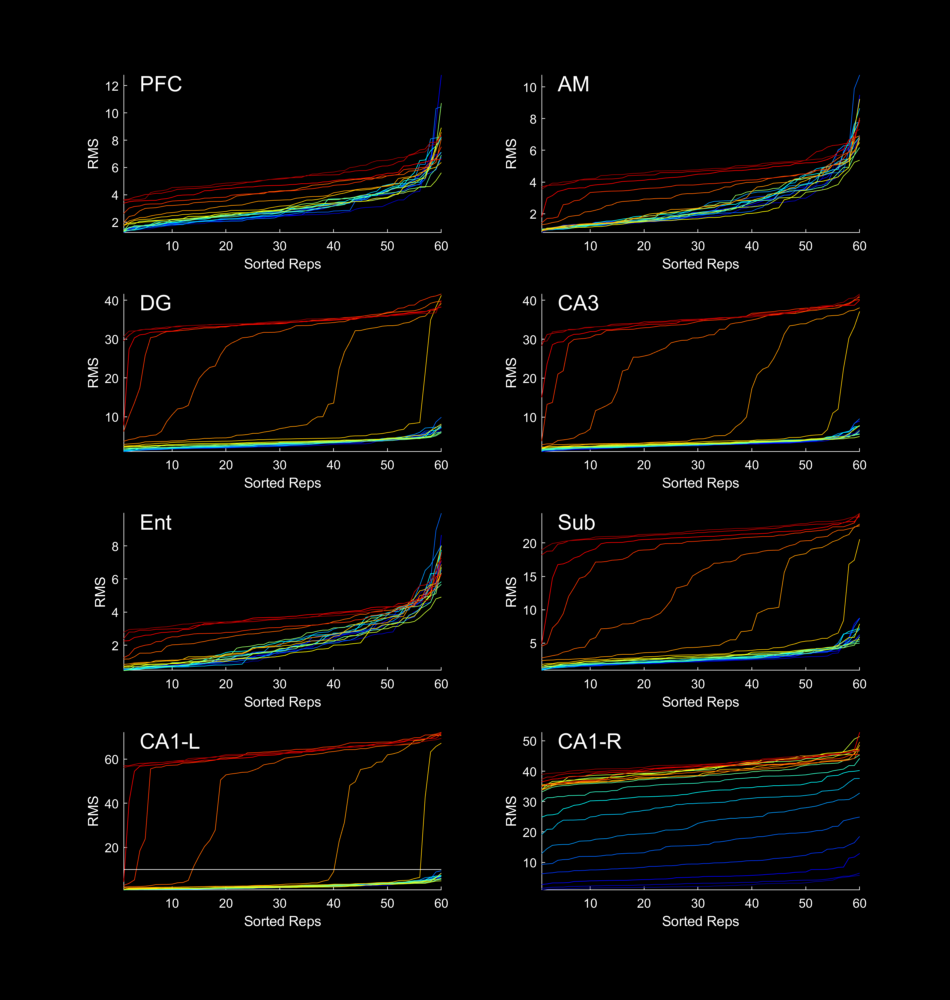

Supplement: Extended data 1 — Supplementary optoDR - example output. Download Extended data 1, ZIP file. [file sup_enu-eN-MNT-0229-18-s01.zip › SID_11-25-15_0003_RMS_sorted_thresh_60_reps.png]

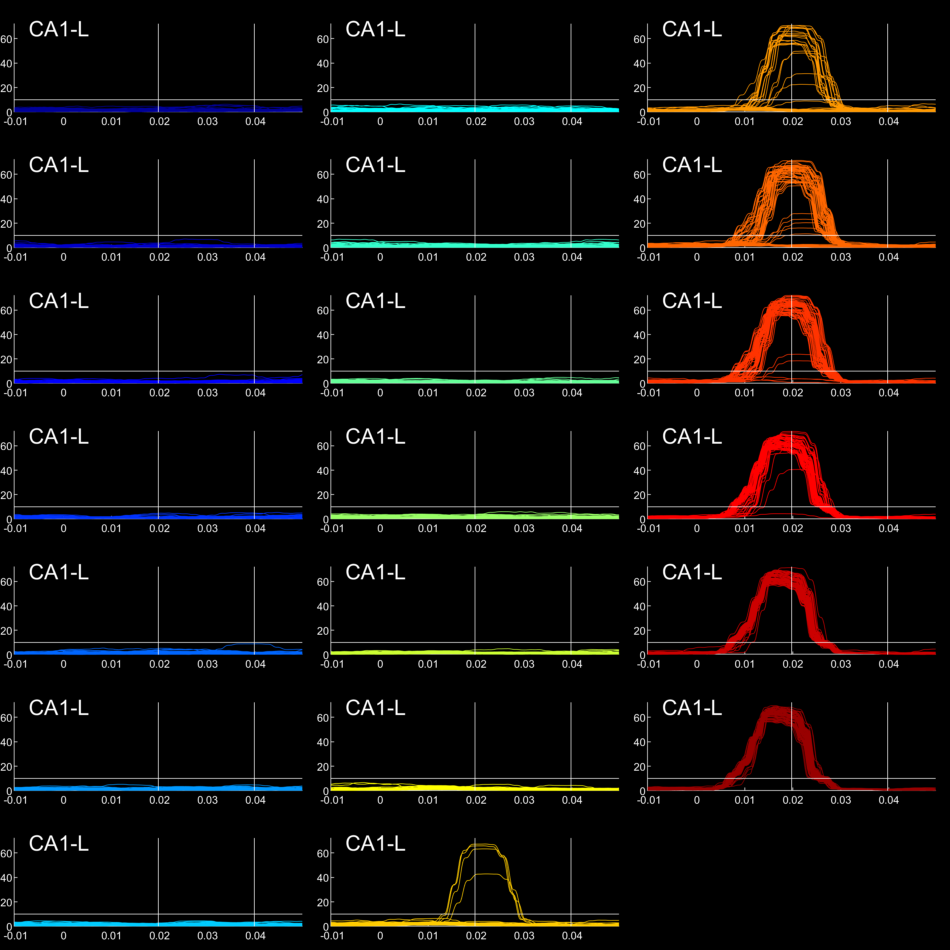

Supplement: Extended data 1 — Supplementary optoDR - example output. Download Extended data 1, ZIP file. [file sup_enu-eN-MNT-0229-18-s01.zip › SID_11-25-15_0003_RMS_thresh_summary_60_reps.png]
